# *Arabidopsis* Carboxylesterase 20 Binds Strigolactone and Increases Branches and Tillers When Ectopically Expressed in *Arabidopsis* and Maize

**DOI:** 10.3389/fpls.2021.639401

**Published:** 2021-04-27

**Authors:** Keith Roesler, Cheng Lu, Jill Thomas, Qingzhang Xu, Peter Vance, Zhenglin Hou, Robert W. Williams, Lu Liu, Michaela A. Owens, Jeffrey E. Habben

**Affiliations:** Corteva Agriscience, Johnston, IA, United States

**Keywords:** strigolactone, carboxylesterase, alpha/beta hydrolase, drought tolerance, *Arabidopsis thaliana*, *Zea mays*, anthesis-silking interval

## Abstract

Severe drought stress can delay maize silk emergence relative to the pollen shedding period, resulting in poor fertilization and reduced grain yield. Methods to minimize the delay in silking could thus improve yield stability. An *Arabidopsis* enhancer-tagged carboxylesterase 20 (*AtCXE20*) line was identified in a drought tolerance screen. Ectopic expression of *AtCXE20* in *Arabidopsis* and maize resulted in phenotypes characteristic of strigolactone (SL)-deficient mutants, including increased branching and tillering, decreased plant height, delayed senescence, hyposensitivity to ethylene, and reduced flavonols. Maize silk growth was increased by *AtCXE20* overexpression, and this phenotype was partially complemented by exogenous SL treatments. In drought conditions, the transgenic maize plants silked earlier than controls and had decreased anthesis-silking intervals. The purified recombinant AtCXE20 protein bound SL *in vitro*, as indicated by SL inhibiting AtCXE20 esterase activity and altering AtCXE20 intrinsic fluorescence. Homology modeling of the AtCXE20 three-dimensional (3D) protein structure revealed a large hydrophobic binding pocket capable of accommodating, but not hydrolyzing SLs. The AtCXE20 protein concentration in transgenic maize tissues was determined by mass spectrometry to be in the micromolar range, well-above known endogenous SL concentrations. These results best support a mechanism where ectopic expression of *AtCXE20* with a strong promoter effectively lowers the concentration of free SL by sequestration. This study revealed an agriculturally important role for SL in maize silk growth and provided a new approach for altering SL levels in plants.

## Introduction

Maize production worldwide ranks first among the cereal crops, but drought stress can significantly limit maize yield (Boyer, 1982). Drought stress during the maize flowering period can substantially delay silk emergence from ear husks while only minimally delaying pollen shed from the tassel (Fuad-Hassan et al., 2008). If the anthesis-silking interval (ASI) from first pollen shed to first silk emergence becomes too great, then poor fertilization, increased ovary abortion, and decreased grain yield can result, as highlighted by multiple studies showing strong inverse correlations of ASI with kernel number and/or grain yield (Fischer et al., 1989; Bolanos and Edmeades, 1996; Campos et al., 2004). Breeding for decreased ASI has been effective in improving maize drought tolerance, and alternatively, breeding for increased yield in the presence of drought stress or stress associated with higher plant density has resulted in decreased ASI (Fischer et al., 1989; Bolanos and Edmeades, 1996; Campos et al., 2004). Still, elite maize hybrids vary widely with respect to ASI (Campos et al., 2004) and further improvements in ASI seem possible (Habben and Schussler, 2017). Silk growth, silk emergence, and silking kinetics have all been proposed as important phenotypes to consider for the improvement of maize drought tolerance (Oury et al., 2016b; Habben and Schussler, 2017).

The α/β hydrolase fold superfamily of proteins includes carboxylesterases, cholinesterases, carbohydrate esterases, lipases, and peptidases (Ollis et al., 1992; Carr and Ollis, 2009). Members of this superfamily contain an 8–11 β-strand core surrounded by connecting α-helices and loops. Their hydrolytic activity is catalyzed by a triad of active site residues consisting of a serine, an aspartate or glutamate, and a histidine. These residues are positioned to form a charge relay system to create a nucleophilic serine capable of attacking the substrate. The structural scaffold of this superfamily is capable of protein-protein interactions, as well as hydrolytic activity, and the evolution of new functions with different interacting partners or substrates is known (Marchot and Chatonnet, 2012). Some members of this superfamily function by binding small molecules without hydrolyzing them, such as the GiD1 protein that functions as a receptor for the plant hormone gibberellin, despite not retaining a complete catalytic triad or hydrolytic activity (Ueguchi-Tanaka et al., 2005).

The carboxylesterase members of this superfamily catalyze the hydrolysis of carboxylesters into alcohol and carboxylate products, and they function in a wide variety of roles across diverse organisms. *Arabidopsis* has 20 carboxylesterase genes that have been phylogenetically organized into seven clades (Marshall et al., 2003). *AtCXE20* is a member of clade II, along with *AtCXE8*, *AtCXE9*, and a carboxylesterase from *Capsicum annuum*, *CaPepEST* (Marshall et al., 2003). No functions have been determined for *AtCXE20* or *AtCXE9*, but *AtCXE8* and *CaPepEst* provide resistance against fungal pathogens (Ko et al., 2005; Lee et al., 2013). The AtCXE20 sequence contains the conserved catalytic serine, aspartate, and histidine residues required for esterase activity, but no activity was observed following expression of recombinant AtCXE20 in *Escherichia coli* (Cummins et al., 2007). Native expression of the *AtCXE20* gene in *Arabidopsis* was observed in all tissues examined, including roots, stems, leaves, flowers, and siliques (Marshall et al., 2003).

Strigolactones (SLs) are β-carotene derived plant hormones (Alder et al., 2012; Al-Babili and Bouwmeester, 2015) that influence shoot architecture by inhibiting branching and tillering (Gomez-Roldan et al., 2008; Umehara et al., 2008). SLs also influence root and root hair growth (Kapulnik et al., 2011), have roles in abiotic stress tolerance (Bu et al., 2014; Ha et al., 2014; Haider et al., 2018), mediate beneficial associations with arbuscular mycorrhizal fungi (Akiyama et al., 2005; Besserer et al., 2006), and are perceived by multiple species of the parasitic weeds *Striga* and *Orobanche* to facilitate their infestation of crops (Conn et al., 2015; Toh et al., 2015; Tsuchiya et al., 2015). The SL perception mechanism is unique among plant hormones, because the SL receptor is an α/β hydrolase fold protein that both perceives the SL signal and can subsequently deactivate SL by hydrolysis (Seto et al., 2019). After binding SL, the receptor undergoes a conformational change to allow interactions with a transcriptional repressor protein and an F box protein component of the E3 ubiquitin ligase complex that degrades the repressor to carry out the SL signal (Jiang et al., 2013; Zhou et al., 2013).

This study demonstrates that strong ectopic expression of an *Arabidopsis* carboxylesterase in maize provides silk phenotypes that are relevant for drought tolerance, most likely *via* a SL sequestration mechanism.

## Results

### Ectopic Expression of *AtCXE20* in *Arabidopsis* Resulted in Increased Branching and Decreased Sensitivity to Ethylene

**Figure 1:**
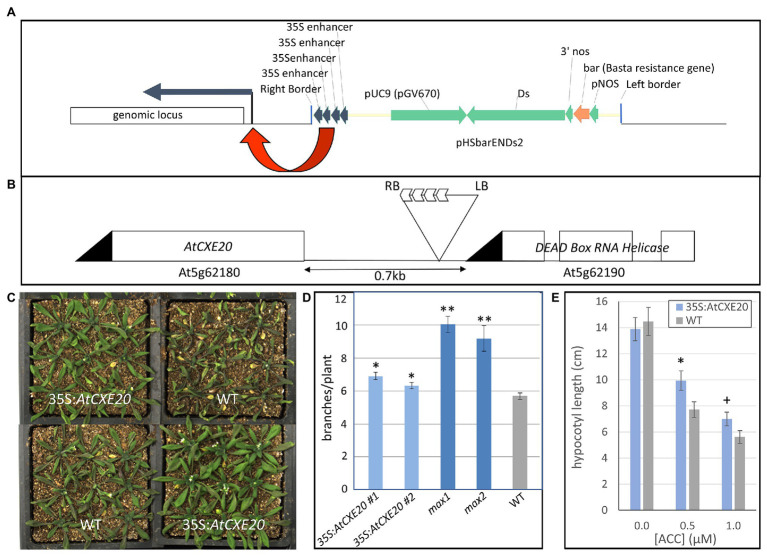
Effects of *AtCXE20* ectopic expression in *Arabidopsis* on branching, sensitivity to ethylene, and retention of green leaf area during drought stress. **(A)** Description of the expression cassette of the T-DNA vector used for creation of the enhancer-tagged library that provided the *AtCXE20* line. Four copies of the cauliflower mosaic virus 35S enhancer were included to increase expression of adjacent genes at the insertion site. **(B)** Insertion locus of the *Arabidopsis AtCXE20* enhancer-tagged line. **(C)** Recapitulation of the drought tolerance phenotype by *AtCXE20* ectopic expression. The CaMV 35S promoter was used to drive *AtCXE20* expression. Greater retention of green leaf area was apparent during drought stress, providing evidence that *AtCXE20* was responsible for the drought tolerance phenotype of the enhancer-tagged line. **(D)** Effect of *AtCXE20* ectopic expression on branching in *Arabidopsis*. The CaMV 35S promoter was used to drive *AtCXE20* expression. 35S:*AtCXE20* #1 and 35S:*AtCXE20* #2 denote two independent single copy events. *max1* and *max2* denote *Arabidopsis* mutants deficient in strigolactone (SL) synthesis and signaling, respectively. WT denotes wild type *Arabidopsis* (ecotype Columbia). Branches were counted on 6-week old plants (*n* = 20). Error bars are SEs. Significant differences from wild type are denoted by single and double asterisks for *p* < 0.01 and *p* < 0.0001, respectively. **(E)** Effect of *AtCXE20* ectopic expression on sensitivity to ethylene. *Arabidopsis* seeds were planted on plates containing media supplemented with the ethylene precursor 1-aminocyclopropane-1-carboxylic acid (ACC) at concentrations of 0, 0.1, and 0.5 μM. The plates were kept in the dark for 4 days at 4°C, followed by 5 days in the dark at 20°C, and then hypocotyl lengths were measured. Error bars are SEs (*n* = 17–21). Significant differences from wild type are denoted by * and + for *p* < 0.05 and *p* < 0.1, respectively.

Activation tagging of *Arabidopsis* with T-DNA vectors containing multiple copies of the cauliflower mosaic virus (CaMV) 35S enhancer is an effective way to increase expression of genes near the insertion site (Weigel et al., 2000). To identify candidate genes that might provide drought tolerance in maize, an *Arabidopsis* library tagged with four copies of the CaMV 35S gene enhancer (Figure 1A) was screened for improved retention of green leaf area during drought stress. In one line with greater leaf area, the 4X enhancer was located between a gene encoding a member of the carboxylesterase gene family, *AtCXE20* (At5g62180), and a gene encoding an RNA helicase (At5g62190; Figure 1B). To determine whether either of these genes was responsible for the phenotype, each gene was separately overexpressed with the CaMV 35S promoter. No phenotypes were observed with overexpression of the RNA helicase, but greater retention of leaf area during drought stress was evident with *AtCXE20* overexpression (Figure 1C). *AtCXE20* overexpression also resulted in increased branching in two independent single copy events (Figure 1D). This increased branching from *AtCXE20* overexpression was less pronounced than that observed with the *Arabidopsis max1* and *max2* mutants that are deficient in SL synthesis and signaling, respectively (Figure 1D). MAX1 encodes a cytochrome P450 that functions in SL synthesis, and MAX2 encodes an F-box protein that functions in SL signaling (Al-Babili and Bouwmeester, 2015). The effect of *AtCXE20* overexpression on sensitivity to ethylene was also examined (Figure 1E). Treatment with increasing concentrations of the ethylene biosynthetic precursor 1-aminocyclopropane-1-carboxylic acid (ACC) resulted in more extreme decreases in hypocotyl growth in wild type *Arabidopsis* compared with transgenic, suggesting that *AtCXE20* overexpression decreased the sensitivity to ethylene.

The TargetP-2.0 program (Emanuelsson et al., 2000) was used to predict targeting of AtCXE20 to different subcellular locations. Liklihoods of 0, 0, 0, 0.0002, and 0.9997 were determined by TargetP-2.0 for mitochondrial transfer peptide, chloroplast transfer peptide, thylakoid luminal peptide, secretory pathway signal peptide, and other, respectively. The lack of targeting predicted for AtCXE20 suggested a cytosolic location. A second subcellular prediction program, WoLF PSORT (Horton et al., 2007) predicted that the most likely subcellular location for AtCXE20 was cytosolic, in agreement with the TargetP-2.0 results.

The *AtCXE20* nucleotide and amino acid sequences are available at NCBI accession NM_125612.

### Ectopic Expression of *AtCXE20* in Maize Increased Tiller Numbers in Field Plots

**Figure 2:**
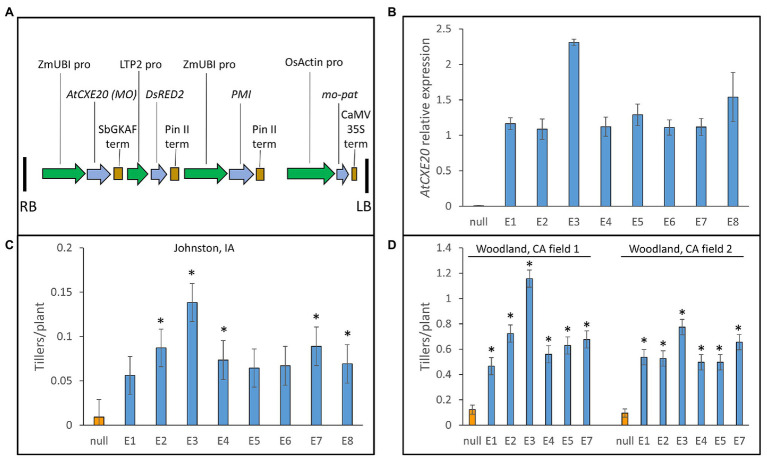
Ectopic expression of *AtCXE20* increases maize tiller numbers in field plots. **(A)** Description of the expression cassette used for *AtCXE20* expression in maize. RB and LB, right border and left border; ZmUBI PRO, the maize ubiquitin1 promoter including the first intron (Christensen et al., 1992); *AtCXE20* (MO), *Arabidopsis thaliana carboxylesterase 20*, optimized for maize codon usage; SbGKAF term, *Sorghum bicolor* gamma kaffirin terminator; LTP2 pro, lipid transfer protein2 promoter; DsRED2, *Discosoma* red fluorescent protein2 color marker; Pin II term, potato proteinase inhibitor II terminator; PMI, phosphomannose isomerase used for selection; OsActin pro, rice actin promoter; mo-pat, maize optimized phosphinothricin acetyltransferase, which provides resistance to the herbicide glufosinolate; CaMV 35S term, cauliflower mosaic virus 35S terminator. **(B)** Relative expression of *AtCXE20* in leaves of eight transgenic events of maize hybrid PHR03 × PH12SG, as determined by quantitative RT-PCR. Error bars are SEs, *n* = 4 field blocks. **(C)** Effect of *AtCXE20* expression on maize tiller numbers at Johnston, Iowa. Error bars are SEs, *n* = 4 field blocks. Significant differences from null are denoted by asterisks, *p* < 0.05. **(D)** Effect of *AtCXE20* expression on maize tiller numbers at Woodland, California. Error bars are SEs, *n* = 4 field blocks. Significant differences from null are denoted by asterisks, *p* < 1 × 10^−5^.

The maize ubiquitin promoter and sorghum gamma kaffirin terminator were used to drive strong constitutive expression of *AtCXE20* in maize (Figure 2A). Seven single-copy transgenic events and a two-copy event (E3) were chosen for a field plot study at Johnston, Iowa using maize hybrid PHR03 × PH12SG. The *AtCXE20* transcript was detected by qRT-PCR in leaves of all events, with the two-copy event E3 having the greatest transcript abundance (Figure 2B). Control plants had only 0.0092 tillers/plant in these typical field plot conditions with high plant density and considerable shading (Figure 2C). Approximately 6–15-fold increases in tiller numbers were observed in the *AtCXE20* transgenic events (Figure 2C). Six of the transgenic events were also studied in plots in two separate fields at the high solar radiation environment of Woodland, California (Figure 2D). In these conditions, control plants had 0.12 and 0.096 tillers/plant in fields 1 and 2, respectively. The *AtCXE20* transgenic events had up to 9.7- and 8.1-fold increases in tiller numbers in fields 1 and 2, respectively. At Johnston and at both Woodland fields, the event with the greatest *AtCXE20* expression (E3) also had the greatest increase in tiller numbers.

### Ectopic Expression of *AtCXE20* Resulted in Earlier Silking and Decreased ASI in Drought-Stressed Conditions in Field Pots

To facilitate outdoor drought stress treatments in a corn belt location that typically lacks severe drought stress, transgenic hybrid maize plants expressing *AtCXE20* were grown outdoors in tree pots supported by wooden racks in 2014 and 2016 in Johnston, Iowa. Drought stresses were then imposed by withholding irrigation and letting pots dry, as described previously (Danilevskaya et al., 2019; Wu et al., 2019). The timing of the drought stress treatments and the natural rainfall occurring during the treatment periods are presented in Supplementary Table 1. The field pot results were analyzed across events (Table 1), and by individual event (Supplementary Table 2). The across-event analysis provided greater statistical power to discern phenotypic differences, while the analysis by individual events allowed assessment of event to event variability.

In water-replete field pot conditions in 2014, the transgenic maize plants had increased tiller numbers and decreased plant height relative to segregating event nulls (Table 1). Tiller numbers for both transgenic and null were greater in pots compared with the plot data of Figure 2, due to the greater spacing resulting in decreased shading of individual plants in pots, compared with a field plot canopy. The transgenic plants also had small decreases in leaf flavonols, and small but equal delays in pollen shedding and silking times, such that the ASI was unchanged. No significant differences in yield, yield components, or harvest index were observed in water-replete conditions (Table 1). The analysis by individual events revealed that the event with greatest *AtCXE20* expression level, E3, had the most pronounced effects on the plant height, tiller number, flavonol, shed, and silk phenotypes in water-replete conditions in 2014 (Supplementary Table 2).

**Table 1:** Effects of *AtCXE20* ectopic expression on hybrid maize grown in the field in pots in water-replete and drought-stressed conditions.

Year	Trait^a^	Water-replete					Drought-stressed				
		Trans	Ctrl	Diff	*p*	Signif	Trans	Ctrl	Diff	*p*	Signif
2014	Tillers/pl	2.67	2.34	0.327	2.47E-05	***	2.72	2.21	0.511	1.46E-09	***
2014	Pl ht. V10 (cm)	86.5	90.4	−3.89	2.35E-09	***	90.7	92.8	−2.15	6.63E-04	***
2014	Pl ht. V16 (cm)	186	191	−5.42	2.37E-09	***	173	174	−0.881	0.285	NS
2014	Flavonols	0.823	0.884	−0.0608	0.0811	+	0.743	0.797	−0.0538	0.0566	+
2014	Chlor (μg/cm2)	44.0	44.7	−0.660	0.449	NS	41.6	41.6	−0.0132	0.985	NS
2014	Shed (DAP)	61.7	61.5	0.236	0.00829	**	62.1	62.1	0.0556	0.749	NS
2014	Silk (DAP)	62.7	62.5	0.236	0.0177	*	65.1	65.7	−0.641	0.0199	*
2014	ASI (days)	1.01	1.00	0.00491	0.967	NS	3.12	3.80	−0.683	0.0273	*
2014	YL/pl	ND^b^	ND	ND	ND	ND	10.8	11.2	−0.439	0.00750	**
2014	Tot DW (g/pl)	412	394	18.3	0.136	NS	217	210	6.86	0.340	NS
2014	Seed DW (g/pl)	193	190	2.42	0.666	NS	91.4	75.9	15.5	0.0137	*
2014	Seed no/pl	623	631	−7.88	0.645	NS	343	279	64.6	0.0121	*
2014	100 seed wt (g)	31.0	30.2	0.768	0.156	NS	27.1	27.1	0.00447	0.994	NS
2014	Harvest index	0.482	0.491	−0.00935	0.236	NS	0.363	0.297	0.0664	0.0334	*
2016	Tillers/pl	2.44	2.44	3.30E-06	0.994	NS	2.53	2.41	0.125	0.213	NS
2016	Pl ht. V9 (cm)	63.3	65.5	−2.20	4.68E-05	***	62.7	64.7	−1.95	0.00288	**
2016	Pl ht. R2 (cm)	243	247	−3.97	0.0109	*	233	241	−7.59	1.85E-07	***
2016	Flavonols	0.920	1.02	−0.101	0.00208	**	0.877	1.02	−0.142	1.44E-06	***
2016	Chlor (μg/cm2)	43.7	44.3	−0.624	0.193	NS	39.5	40.9	−1.35	0.0160	*
2016	Shed (DAP)	53.0	53.4	−0.365	0.00368	**	53.4	54.1	−0.642	1.78E-04	***
2016	Silk (DAP)	53.8	54.2	−0.419	0.0277	*	54.2	55.4	−1.21	1.50E-09	***
2016	ASI (days)	0.736	0.780	−0.0384	0.868	NS	0.720	1.36	−0.635	1.49E-04	***
2016	Tot DW (g/pl)	305	344	−39.0	2.30E-06	***	276	276	0.426	0.927	NS
2016	Seed DW (g/pl)	140	156	−15.9	5.31E-04	***	129	125	3.97	0.354	NS
2016	Seed no/pl	393	423	−30.9	0.00993	**	361	335	26.3	0.0694	+
2016	100 seed wt (g)	0.611	0.610	8.91E-04	0.251	NS	35.9	35.4	0.467	0.449	NS
2016	Harvest index	0.463	0.450	0.0115	0.206	NS	0.468	0.433	0.0345	0.00876	**

Hybrid maize plants were grown outdoors in 2014 and 2016 at Johnston, Iowa in irrigated pots supported by wooden racks. Fifteen and 18 replications were used in 2014 and 2016, respectively. The 2014 experiment used hybrid PHR03 × PH12SG and had seven sequential drought stresses imposed before and during flowering. Two single-copy transgenic events (E1 and E2) and one two-copy event (E3) were studied in 2014, with segregating event nulls used as controls. The 2016 experiment used hybrid PH184C × PH1V69 and had only four sequential drought stresses imposed. Three single-copy events (E9, E10, and E11) were studied in 2016, with wild type plants used as controls. The across event analysis is presented here, and the analysis by individual events is presented in Supplementary Table 2. Statistically significant differences between transgenic and control within an environmental treatment are denoted by ***, **, *, and +, for value of *p* < 0.001, 0.01, 0.05, or 0.1, respectively. Trans, transgenic. Ctrl, controls.

a Pl ht., plant height; Tillers/pl., number of tillers per plant; Flavonols, leaf flavonols index; Chlor, leaf chlorophyll; Shed and Silk, days after planting for first pollen shed and first silk emergence; ASI, anthesis-silking interval; YL/pl., number of yellow leaves (>50% yellow) per plant; DW, total aboveground dry weight per plant at R6; Seed DW, seed yield defined as dry weight of total kernels per plant at R6; Seed no/pl., kernel number per plant at R6; 100 seed wt, dry weight of 100 kernels at R6. Harvest index was defined as seed dry weight divided by total above ground dry weight.

b ND, not determined.

In drought-stressed conditions in 2014, transgenic maize plants had increased tiller numbers, decreased plant height, and decreased leaf flavonols compared with null controls (Table 1). Both later pollen shedding and earlier silking were observed in transgenic plants, resulting in a significant decrease in ASI. The decreased ASI allowed more effective pollination in these severe drought conditions, resulting in significantly increased seed number, seed yield, and harvest index compared with nulls. No differences in total above-ground dry weight or in 100-seed weight were observed. Delayed senescence, as measured visually by a decrease in yellow leaves, was also observed in transgenic plants. The analysis by individual events revealed that significantly increased tillers, decreased flavonols, delayed senescence, and increased seed number were observed in multiple events in the 2014 drought stressed conditions (Supplementary Table 2). However, the earlier silking, decreased ASI, and increased seed dry weight and harvest index phenotypes were statistically significant only with the highly expressing event E3.

In the across-event analysis of the 2016 field pot study (Table 1), decreased plant height, decreased flavonols, earlier shedding, and earlier silking were observed in transgenic plants in both environments, but increased tillering was not significantly different between transgenic and control plants. In water-replete conditions, decreases in total dry weight, seed number, and seed dry weight (yield) were apparent. In drought conditions, transgenic plants had earlier silking and shedding, decreased ASI, increased seed number, and increased harvest index. In the analysis by individual events for the 2016 study (Supplementary Table 2), the early silking phenotype in drought conditions was especially pronounced, with all three transgenic events having highly significant differences in silking time of more than a full day earlier than controls, resulting in significantly decreased ASI for two of the events.

To summarize the field pot studies, ectopic expression of *AtCXE20* in maize provided earlier silking and decreased ASI in drought conditions across two hybrid genotypes. Although these are agriculturally valuable phenotypes, the strong constitutive expression of *AtCXE20* with the ubiquitin promoter was clearly not optimal, resulting in decreased total biomass and seed yield in 2016 in water-replete conditions.

### AtCXE20 Esterase Activity Was Inhibited by Strigolactones, but Not by Other Hormones

**Figure 3:**
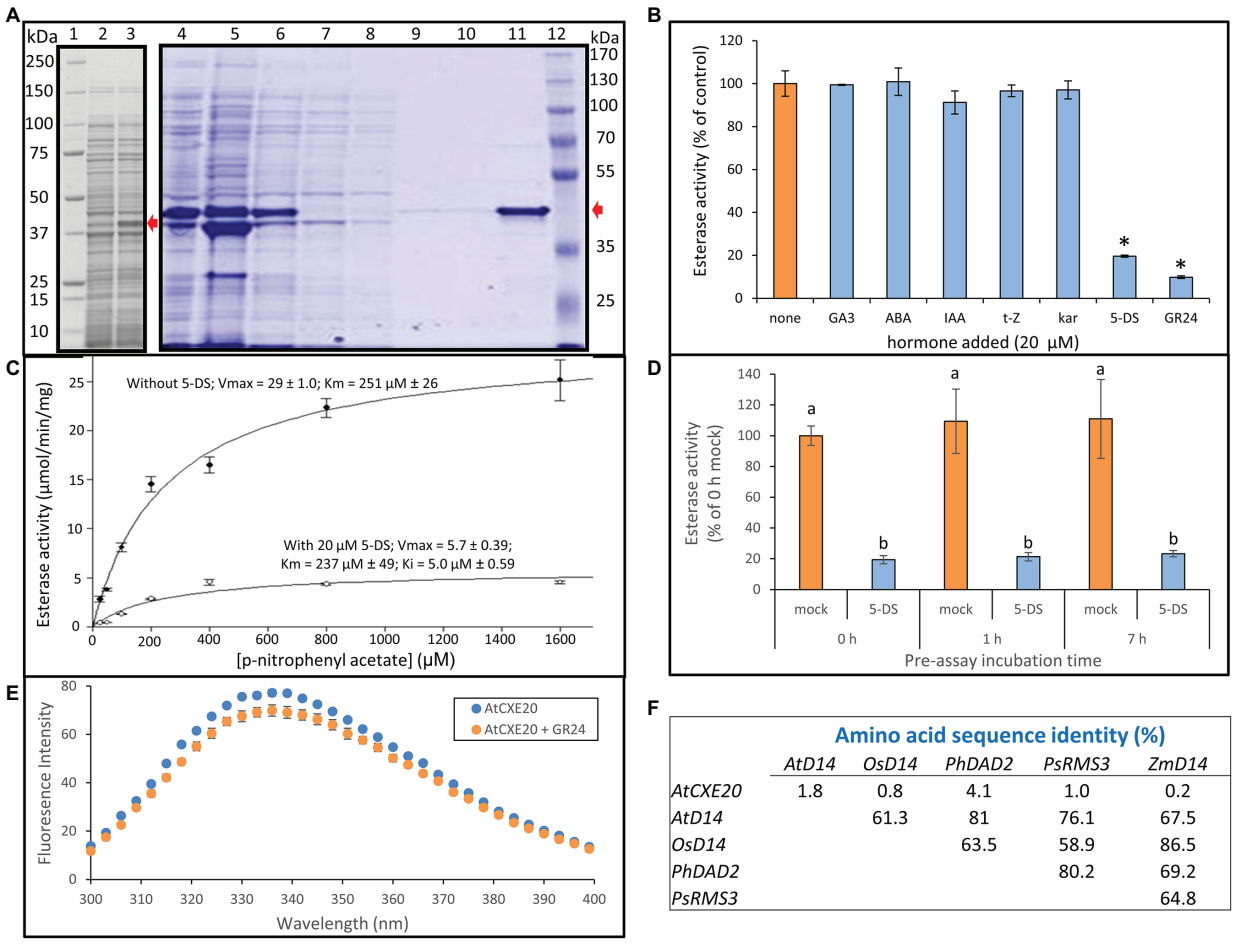
Inhibition of AtCXE20 esterase activity by strigolactones. **(A)** SDS-PAGE analysis of recombinant AtCXE20 protein used for esterase assays. AtCXE20 with a 6-HIS N-terminal tag was expressed in *Escherichia coli* and purified by cobalt affinity chromatography. Lanes 1 and 12, molecular mass markers; Lane 2, uninduced culture; Lane 3 induced culture; Lane 4, whole cell lysate; Lane 5, insoluble protein; Lane 6, soluble protein; Lane 7, unbound protein; Lanes 8–10, washes; Lane 11, eluted AtCXE20 protein. Red arrows mark the AtCXE20 position in each gel. **(B)** Effect of 20 μM hormone addition on AtCXE20 carboxylesterase activity, expressed as a percent of the control value with no added hormone. Assays were done with 800 μM p-nitrophenyl acetate as substrate. Hormones were GA3, gibberellic acid-3; ABA, abscisic acid; IAA, indole-3-acetic acid; t-Z, trans-zeatin; kar, karrikin-3; 5-DS, 5-deoxystrigol; GR24, *rac*-GR24. Error bars are SEs, *n* = 3 except for GA3, ABA, and IAA, for which *n* = 2. Significant differences from the no hormone control are denoted by asterisks, *p* < 0.001. **(C)** Determination of AtCXE20 kinetic parameters in the presence or absence of 20 μM 5-DS, using p-nitrophenyl acetate as substrate. Inhibition by 5-DS was noncompetitive, with a Ki of 5 μM, as determined by the Sigmaplot enzyme kinetics module. Error bars are SEs, *n* = 3. **(D)** Effect of pre-assay incubation time on AtCXE20 esterase activity and extent of inhibition by 5-DS. Purified AtCXE20 recombinant protein was incubated for 0, 1, or 7 h at 4°C with 20 μM 5-DS or an equal volume of DMSO, prior to assays at 25°C. Error bars are SEs, *n* = 3. Significant differences are denoted by different letters, *p* < 0.05. **(E)** Effect of strigolactone on AtCXE20 intrinsic fluorescence. An excitation wavelength of 280 nm was used. Measurements were done in quartz cuvettes with 1 μg of AtCXE20 in 500 μl of 10 mM sodium phosphate, pH 7, in the presence of 10 μM GR24 or an equal volume of DMSO. Emission spectra were corrected by subtracting values for buffer blanks containing GR24 or DMSO, but without AtCXE20. Error bars are SEs, *n* = 3. Significant differences in the presence vs. the absence of GR24 (*p* < 0.05) were determined for wavelengths 300, 306, 315, 318, 321, 324, 327, 330, 333, 339, 342, 354, 366, and 393 nm. **(F)** Amino acid sequence identities of AtCXE20 and well-characterized strigolactone receptors. The method of Needleman and Wunsch (1970) was used, with a gap creation penalty of 8 and a gap extension penalty of 2. Gene names and NCBI reference numbers are: AtD14, *Arabidopsis thaliana* D14, NP_566220; OsD14, *Oryza sativa* D14, NP_001050445; PhDAD2, *Petunia hybrida* DECREASED APICAL DOMINANCE 2, J9U5U9; PsRMS3, *Pisum sativum* RAMOSUS3, KT321518; ZmD14, *Zea mays* D14, XM_008662207.

In a previous study, the SL receptor from *Pisum sativum*, RAMOSUS3, displayed Michaelis-Menten kinetics for esterase activity using the synthetic substrate p-nitrophenyl acetate (pNPA), and the esterase activity was inhibited by addition of SL (de Saint Germain et al., 2016). The purified recombinant AtCXE20 protein was similarly examined for inhibition of esterase activity by hormones, using pNPA as substrate (Figure 3). A considerable fraction of the recombinant protein was soluble in *E. coli* lysates and was amenable to purification by immobilized metal affinity chromatography (Figure 3A). This N-terminally tagged AtCXE20 protein had esterase activity, in contrast to a C-terminally tagged AtCXE20 that lacked activity in a previous study (Cummins et al., 2007). No inhibition of AtCXE20 esterase activity was observed with gibberellin, abscisic acid, auxin, cytokinin, or karrikin (Figure 3B). However, substantial inhibition was observed with the synthetic SL racemic mixture GR24 that is frequently used for exogenous supplementation in SL studies, and with a pure (+) isomer of the naturally-occurring SL 5-deoxystrigol (5-DS) that was previously detected in root exudates of maize (Awad et al., 2006; Yoneyama et al., 2015). The more abundant zealactones recently discovered in maize root exudates (Charnikhova et al., 2017) were not commercially available. The substantial inhibition of esterase activity by SLs appeared to be due to SL binding to the AtCXE20 protein, rather than binding to the substrate, because the substrate concentration of 800 μM was 40 times greater than the SL concentration of 20 μM. Therefore, even if all of the SL had bound 1:1 with substrate molecules, there would have been only a minor reduction in substrate concentration available to the enzyme, resulting in little effect on activity. The AtCXE20 esterase activity followed Michaelis-Menten kinetics. In the absence of 5-DS, the AtCXE20 Vmax for esterase activity was 29 ± 1.0 μmol/min/mg protein, and the Km was 251 ± 26 μM (Figure 3C). In the presence of 20 μM 5-DS, the Vmax was 5.7 ± 0.39 μmol/min/mg protein, and the Km was 237 ± 49 μM. The significant decrease in Vmax, and no significant change in the Km value showed that the inhibition by 5-DS was noncompetitive. The 5-DS inhibition constant (Ki) was determined by Sigmaplot software to be 5 μM (Figure 3C). Noncompetitive inhibition indicated that the substrate and the hormone bound to AtCXE20 without overlap. If they had bound at the same or overlapping sites, then competitive inhibition would have been expected, with a larger Km but a similar Vmax because substrate at high concentrations would outcompete the inhibitor for the binding site.

In cases where an inhibitor permanently inactivates an enzyme by covalent modification, preincubating the enzyme with the inhibitor leads to increasingly severe inhibition with time (Rome and Lands, 1975). Pre-incubating AtCXE20 with 5-DS for up to 7 h prior to esterase assays did not significantly further inhibit AtCXE20 esterase activity (Figure 3D), providing evidence that AtCXE20 is not permanently inactivated by covalent modification with 5-DS. The presence of 5-DS appeared to stabilize the activity of the diluted AtCXE20 protein over time, as indicated by the smaller SE bars observed in the presence of 5-DS compared with mock treatments having no 5-DS but an equal volume of DMSO (Figure 3D).

Esterase assays were also attempted with p-nitrophenyl butyrate as substrate. The recombinant AtCXE20 protein was inactive with this slightly larger substrate that had an acyl chain length only two carbons longer than that of pNPA.

The effect of SL on the intrinsic fluorescence of AtCXE20 was also examined. In the absence of GR24, the AtCXE20 fluorescence emission spectrum had a broad peak with a maximum value at 336 nm (Figure 3E). In the presence of 10 μM GR24, there was a small decrease in AtCXE20 intrinsic fluorescence, but no pronounced blue or red shifts were evident. The decreased fluorescence was further evidence that AtCXE20 can bind SL *in vitro*.

Although AtCXE20 and SL receptors share an α/β hydrolase protein fold, AtCXE20 had almost no amino acid sequence identity with the well-characterized SL receptors from *Arabidopsis*, rice, pea and petunia, or with the putative maize ortholog (GRMZM2G008751; Figure 3F).

### Homology Modeling of the AtCXE20 3D Structure Revealed a Large Hydrophobic Cavity Capable of Accommodating, but Not Hydrolyzing SLs

**Figure 4:**
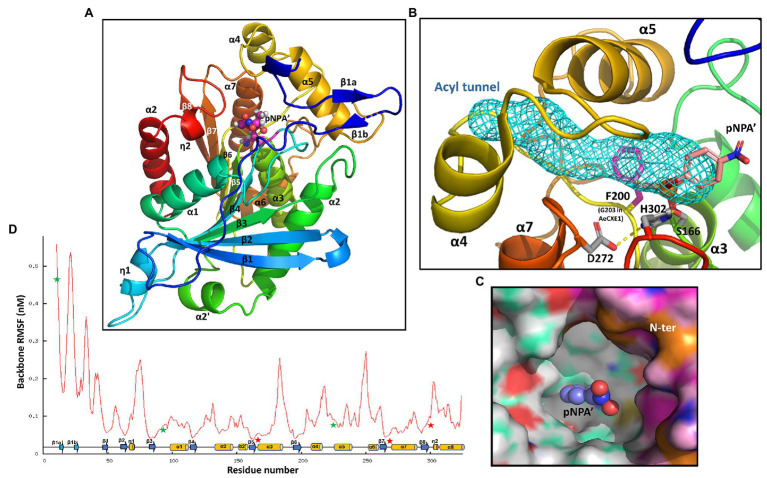
AtCXE20 three-dimensional modeling. **(A)** A cartoon representation of the overall AtCXE20 structure, with the peptide chain being spectral colored from N-terminal blue to C-terminal red. The secondary structural elements are labeled with α for helices, β for strands, and η for 3_10_ helices. Stick-balls atop the β5 C-terminal end denote an adduct of S166 and 4-nitrophenyl methylphosphonate (4NMP), a transition state mimic of the artificial substrate used here for activity assays, p-nitrophenyl acetate. This transition state mimic is labeled pNPA’. **(B)** Potential substrate acyl-group tunnel blockage by F200 in AtCXE20. Sticks represent F200 and the AtCXE20 catalytic triad residues S166 adducted with 4NMP, H302, and D272. The mesh void surrounded by α5, α6, and linking loops is the substrate acyl-group tunnel of AeCXE1. F200 in AtCXE20 corresponds to G203 in AeCXE1, and the presence or absence of a large side chain at this position results in tunnel blockage in AtCXE20, but not in AeCXE1. **(C)** The large hydrophobic AtCXE20 substrate binding cavity. The near N-terminal end composing part of the cavity rim is colored magenta. The balls represent the adducted 4NMP. **(D)** Average backbone fluctuations along the AtCXE20 polypeptide chain during a 35-nanosecond molecular dynamic (MD) simulation. Arrows and cylinders depict the secondary structural elements for β strands and α helices, respectively. Stars mark the substrate binding regions, with red stars indicating the catalytic triad location.

The noncompetitive inhibition of AtCXE20 activity by 5-DS suggested non-overlapping binding sites for 5-DS and the pNPA substrate. To determine whether the AtCXE20 binding pocket was large enough to accommodate both 5-DS and pNPA without overlap, homology modeling of the AtCXE20 3D structure was done based on the known 3D structure of *Actinidia eriantha* (kiwifruit) carboxylesterase 1 (AeCXE1; Ileperuma et al., 2007). The AtCXE20 amino acid sequence had 51% identity and 78% similarity compared with that of AeCXE1 (pdb:2o7v), with only a few short indels occurring on surface loops. The AtCXE20 homologous model was generated by MODELER on Accelrys Discovery Studio. Due to the high sequence similarity with the structural template, the resulting model had a high quality with a normalized DOPE (discrete optimized energy) score of −1.66, significantly lower than the native-like model threshold score of <−1. As observed in other α/β-hydrolases, the AtCXE20 structure consisted of a central eight-stranded β-sheet with flanking α-helices on both sides, forming a regular succession of α-helices and β-strands (Figure 4A). The protein structure placed all the essential catalytic elements in proper positions for carboxylester hydrolysis. The conserved nucleophilic residue S166 in the pentapeptide GxSxG motif was located on top of a sharp turn between β5 and α3, known as the “nucleophile elbow.” The unusually strained nucleophile elbow activates the S166 Oγ for nucleophile attack. The catalytic triad residues S166, H302 at the β8-α8 loop, and D272 at the β7-α7 loop, were all in hydrogen bonding distances suitable for shuttling a proton and facilitating the hydrolysis (Figure 4B). The “oxyanion hole” was comprised of G89 and G90 on the β3-α1 loop, and A167 following the nucleophile S166, all surrounding the catalytic center. Their peptide amide -NH groups were capable of hydrogen bonding the substrate carboxyl oxygens to stabilize the transition state.

Although AeCXE1 and AtCXE20 have similar catalytic mechanisms, they differ greatly in the sizes of their hydrolysable substrates. AeCXE1 can hydrolyze 4-methylumbelliferyl esters with acyl chain lengths varying from 2-carbon acetyl chains to 16-carbon palmityl chains (Ileperuma et al., 2007), while AtCXE20 can hydrolyze only p-nitrophenyl acetate with a 2-carbon acetyl chain but not p-nitrophenyl butyrate with a 4-carbon acyl chain (this study). Both proteins have a funnel-shaped deep gorge responsible for binding the substrate, with the catalytic triad and oxyanion hole located at the bottom (Figure 4B). In the unliganded AeCXE1 structure, the deep gorge is extended by a long and narrow hydrophobic tunnel surrounded by α4, α5, and their connecting loops (Figure 4B). This tunnel is in the correct orientation to harbor a long 4-methylumbelliferyl acyl carbon chain. In the AtCXE20 structure, however, the benzene ring of F200 directly inserts into the acyl tunnel, shortening it substantially (Figure 4B). This single amino acid difference, F200 in AtCXE20, corresponding to G203 in AeCXE1, is likely responsible for the observed differences in substrate acyl chain lengths for these enzymes. The AtCXE20 F200 is located immediately after β6, and it is near the nucleophilic serine.

Based on the AeCXE1 crystal structure acyl adduct (diethyl phosphate attached to the nucleophilic serine), we built a model of 4-nitrophenyl methylphosphonate covalently bound to S166, a transition state mimic of the substrate used here for AtCXE20 activity assays, p-nitrophenyl acetate. The model showed that all the polar atoms around the catalytic center properly form hydrogen bonds with -NHs of “oxyanion hole” residues and with the catalytic triad residue H302. The model revealed that the acetyl methyl group of this transition state mimic forms a direct Van der Waals contact with the F200 benzene ring, suggesting that any potential substrates with acyl groups larger than an acetyl group, including p-nitrophenyl butyrate and strigolactones, would be unlikely to be hydrolyzed by AtCXE20. Larger acyl groups would move the ester bond position of an ester substrate, or the ether linkage of SLs, further away from the AtCXE20 catalytic triad. Although such substrates may bind to the hydrophobic gorge, their larger acyl groups would not allow sufficient proximity and orientation to the catalytic triad to allow efficient hydrolysis.

As observed previously with AeCXE1, the AtCXE20 substrate binding gorge is deep, hydrophobic, and wide open and its walls are made of flexible loops including the α4-α5 loop, the β8-α8 loop, and the N-terminal loop (Figure 4C). The gorge is large enough to accommodate strigolactones and similarly sized hydrophobic molecules in the absence or presence of p-nitrophenyl acetate. To further explore the gorge’s flexibility, we carried out a 35-nanosecond molecular dynamic (MD) simulation with the AtCXE20 unliganded model structure using GROMACS. Figure 4D shows the average root means square fluctuation of the backbone atoms. As expected, the central β sheet and packing helices show strong stability while the loops and N-terminus, including the gorge elements, fluctuate drastically. It is conceivable that upon binding of hydrophobic ligands such as strigolactones, the flexible gorge rim can fold in to make a binding “snag.” A similar hydrophobic cavity with almost identical geometric location was found in the rice GID1 structure that functions in gibberellin binding and signaling (Ueguchi-Tanaka et al., 2005). Compared with AtCXE20, GID1 has an extra extension of two helices at the N-terminus. As seen with the AtCXE20 N-terminus (Figure 4D), the GID1 N-terminal extension is flexible and disordered in the unliganded form. When gibberellin binds to the cavity, the N-terminal extension assumes two helices and covers the cavity as a lid, enabling interaction with the DELLA protein.

### Maize Silk Growth *in vitro* Was Increased by *AtCXE20* Overexpression and Decreased by Exogenous SL Treatments

**Figure 5:**
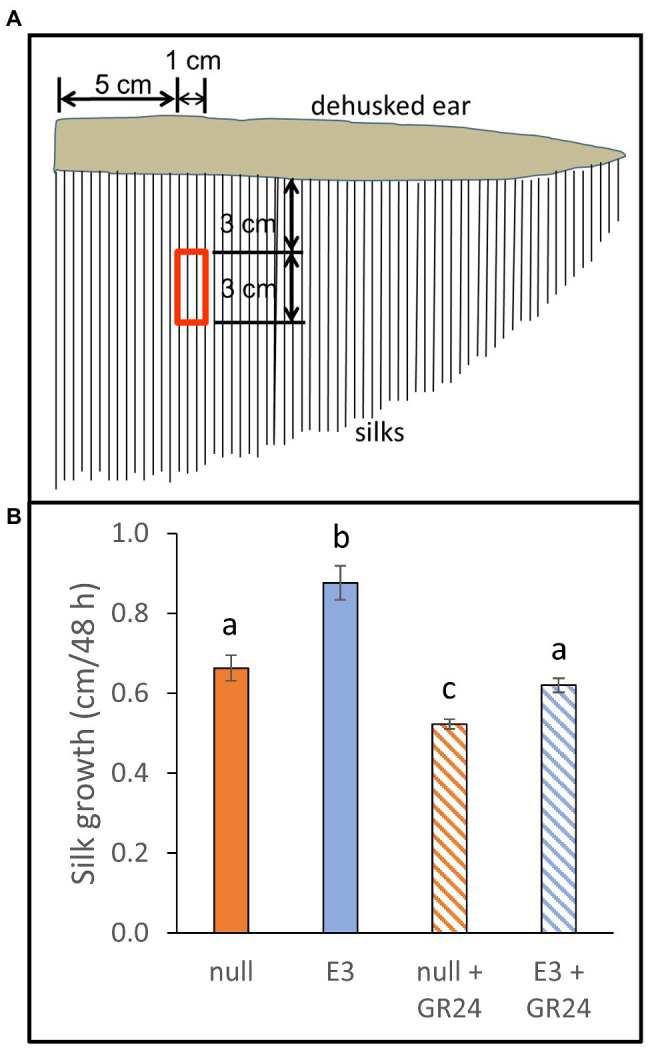
Effects of *AtCXE20* ectopic expression and strigolactone treatment on maize silk growth *in vitro*. **(A)** Description of silk samples. At 1 day after first silk emergence, husks were removed and silk sections of 3 cm in length were excised at the location corresponding to the red boxed area, 5–6 cm from ear base, and 3–6 cm from ear surface. Silk samples were obtained from hybrid PHR03 × PH12SG transgenic event E3 and from segregating event null plants. **(B)** Effects of exogenous GR24 treatment on silk growth *in vitro*. Silk samples were incubated in 1 X MS media, 1% sucrose, 50 μg/ml kanamycin sulfate, and 10 μM GR24 or mock for 48 h, and then silk lengths were measured manually. Error bars are SEs, *n* = 3 ears, with 10 silks measured per ear. Significant differences are denoted by different letters, *p* < 0.05.

Silks from transgenic and null plants of maize hybrid PHR03 × PH12SG were grown *in vitro* in the presence or absence of GR24 (Figure 5). In the absence of GR24, transgenic silks grew faster than control silks. Growth rates of both transgenic and null silks were substantially reduced by the GR24 treatment, providing evidence that SL signaling was still functional in transgenic silks. The GR24 treatment diminished the transgenic silk growth phenotype, with transgenic silk growth being 0.21 cm and 32% greater than nulls in the absence of GR24, but only 0.097 cm and 18% greater than nulls in the presence of GR24.

### The AtCXE20 Protein Concentration Was in the Micromolar Range in Transgenic Maize Tissues

To determine the quantity of AtCXE20 protein in transgenic maize tissues, liquid chromatography coupled with tandem mass spectrometry was done (Table 2). Use of the strong maize ubiquitin promoter resulted in AtCXE20 protein levels ranging from 1,202 to 2,482 ppm, or about 0.12 to 0.25% of the leaf protein, and 355 to 823 ppm, or about 0.036 to 0.082% of the root protein. The *in vivo* concentration of total protein across diverse prokaryotic and eukaryotic cell types averages about 200 mg per ml of cell volume (Albe et al., 1990). This value, the mass spectrometry data for AtCXE20 as a proportion of the total protein, and the AtCXE20 molecular mass of 36,180 daltons were used to convert the concentrations of AtCXE20 protein to a molar basis. In both leaves and roots, the AtCXE20 protein concentrations were in the low micromolar range (Table 2). These AtCXE20 protein concentrations were substantially greater than SL concentrations in plant tissues which are thought to be in the picomolar to nanomolar range, as indicated by less than 20 and 0.4 pmol per gram fresh weight of *Lotus japonicus* roots and shoots, respectively, (Liu et al., 2015), and less than 8 pmol per gram fresh weight of roots from multiple species (Flokova et al., 2020).

**Table 2:** Quantitative determination of AtCXE20 protein in transgenic maize tissues.

Event	Tissue	AtCXE20 protein (ppm)	AtCXE20 protein (μM)
E3	Leaf	2,482 ± 43	13.7
E2	Leaf	1,285 ± 24	7.1
E1	Leaf	1,202 ± 4.9	6.6
E3	Root	823 ± 47	4.6
E2	Root	355 ± 22	2.0

The abundance of AtCXE20 protein in transgenic maize leaves and roots was determined by liquid chromatography coupled with tandem mass spectrometry and expressed as parts per million of protein (mean ± SE, *n* = 3). The AtCXE20 protein concentrations were then converted to a molar basis as described in the results section. Maize hybrid PHR03 × single copy events E1 and E2, and 2-copy event E3 were sampled.

## Discussion

The ability of AtCXE20 to bind strigolactones *in vitro* was consistent with the phenotypes observed in *AtCXE20*-overexpressing *Arabidopsis* and maize plants. The increased tillering/branching and decreased plant height phenotypes observed here are characteristic of plants deficient in SL synthesis or signaling (Gomez-Roldan et al., 2008; Umehara et al., 2008; Guan et al., 2012). The delayed senescence and decreased sensitivity to ACC observed here are consistent with a previous report that SL promotes senescence by enhancing the action of ethylene, and that SL signaling mutants are ethylene hyposensitive (Ueda and Kusaba, 2015). The reduction in leaf flavonols observed here also agreed well with previous reports that an *Arabidopsis* mutant deficient in SL signaling had decreased protein levels for flavonol pathway enzymes in roots, and that exogenous SL treatments increased the protein levels of these enzymes in wild type *Arabidopsis* roots but not in roots of the SL signaling mutant (Walton et al., 2016). *Arabidopsis* plants deficient in SL signaling also had decreased expression in leaves of many genes involved in flavonoid synthesis, including a flavonol synthase (Ha et al., 2014). The improved retention of green leaf area in *AtCXE20*-overexpressing *Arabidopsis* plants during drought stress was consistent with some, but not all, of the previous studies investigating the complicated effects of SL on drought tolerance. The rice SL and karrikin signaling deficient mutant *d3*, and the SL biosynthesis deficient mutants *d17* and *d10* (although not d27) had improved drought tolerance as demonstrated by increased survival of drought treatments and decreased rate of water loss in detached flag leaves (Haider et al., 2018). The *Arabidopsis* SL and karrikin signaling deficient mutant *max2*, and the SL biosynthesis deficient mutants *max3* and *max4* were less drought tolerant as shown by decreased survival of drought treatments, decreased relative water content, and ABA hyposensitivity in germination tests (Ha et al., 2014), but another *Arabidopsis* study concluded that only *max2* was drought susceptible, and that *max3* and *max4* were similar to wild type with respect to survival of drought treatments, the rate of water loss, and sensitivity to ABA or osmotic stress (Bu et al., 2014). These previous studies provided evidence for the involvement of SL in response to drought treatments, but because their results vary so widely, they cannot all be in agreement with the present study. The effects of SL on maize silking and ASI were not reported previously, so the silk-related phenotypes observed here cannot yet be compared with other SL studies.

Collectively, the results of this study best support a model where AtCXE20 binds to and sequesters SL in transgenic plants, effectively lowering the concentration of free SL. Exogenous SL treatments gave a clear phenotype in transgenic maize silks, demonstrating that SL signaling was still functional and suggesting that loss of SL signaling was a less likely mechanism compared with SL sequestration. Homology modeling of the AtCXE20 3D structure revealed that AtCXE20 was not capable of efficiently hydrolyzing SL, suggesting that SL hydrolysis was a less likely mechanism compared with SL sequestration. The micromolar concentrations of AtCXE20 protein determined here in transgenic maize tissues, together with the 5 μM value determined for the 5-deoxystrigol Ki (the dissociation constant of the inhibitor) suggested that AtCXE20 would have sufficient abundance and binding affinity to sequester appreciable amounts of at least some SLs in transgenic tissues. The complementation of the enhanced silk growth phenotype by exogenous SL treatments was also consistent with a SL sequestration mechanism. Finally, the observation that the increased branching in *AtCXE20*-overexpressing *Arabidopsis* was less pronounced than that observed in SL synthesis or signaling mutants was also consistent with a sequestration mechanism that would only reduce the concentration of free SL, but would not completely eliminate SL synthesis or signaling.

There is precedent for a carboxylesterase gain of function resulting in sequestration of a small molecule. A gene amplification mechanism in *Myzus persicae* (peach-potato aphid) increased the abundance of a carboxylesterase that binds carbamate and organophosphate insecticides (Field et al., 1988), such that the carboxylesterase protein comprised 3% of the total insect protein (Devonshire and Moores, 1982). Although this carboxylesterase had poor hydrolytic activity against carbamates, its high protein abundance resulted in sequestration of the insecticide to allow resistance (Devonshire and Moores, 1982). These two carboxylesterase examples illustrate that any mechanism that significantly increases the concentration of an α/β hydrolase fold protein could potentially result in sequestration of a small molecule, even if the mechanism does not improve binding affinity or hydrolytic activity. In the present study, the increased protein concentration was achieved by strong ectopic expression of a transgene, and in the insect example, by amplification of gene copy number, but mutations in the promoter region of an α/β hydrolase gene to increase transcription, or in the coding region to increase protein stability, RNA stability, or translation efficiency could achieve a similar outcome.

A negative role for SL in maize silk growth was revealed by this study, because exogenous SL applications decreased the growth rate of both transgenic and control silks, and because *AtCXE20* transgenic plants with decreased free SL concentrations had earlier silk emergence in the field and increased silk growth rate *in vitro*. Silk growth prior to pollen shed is due to both cell division and tissue expansion, but silk cell division ceases at pollen shed, and subsequent silk growth during the ASI period is due entirely to tissue expansion (Fuad-Hassan et al., 2008). The field results showing a decreased ASI period due to earlier silk emergence, and the *in vitro* results obtained with silks sampled after the start of pollen shed, both indicated that SL negatively affected silk expansive growth. Decreases in silk expansive growth from drought stress were previously associated with altered expression of silk pectinases and expansins (Oury et al., 2016a). It will be interesting to determine whether decreased SL can mitigate these changes in silk gene expression during stress. The early silking and decreased ASI phenotypes observed here are potentially valuable for increasing grain yield if they can be recreated using tissue-specific or drought-inducible *AtCXE20* expression, rather than constitutive expression, to avoid the yield penalty observed in water-replete conditions with one of the two genotypes tested.

Strigolactones influence plant architecture, a key determinant of crop yield (Wang et al., 2018). For example, rice alleles with decreased expression of the SL biosynthesis gene *D17* contributed to improved plant architecture during the green revolution (Wang et al., 2020). Altering SL synthesis or signaling, therefore, has the potential to improve crops, but such alterations may need to be done in a partial or tissue-specific manner to avoid deleterious phenotypes previously observed with constitutive, complete knockouts of SL-related genes. For example, the maize *ccd8* mutant that is deficient in SL biosynthesis had decreased ear size, root growth, and stalk diameter (Guan et al., 2012). Ectopic expression of *AtCXE20* to sequester SL in plant tissues may offer some advantages over constitutive SL knockouts. Tissue-specific promoters could be used to drive *AtCXE20* expression in only the desired tissues, such as silks and/or other reproductive tissues in maize, while avoiding deleterious phenotypes such as reduced root growth. Furthermore, AtCXE20 could sequester SL in the tissue of choice regardless whether the SL was synthesized in that tissue or was synthesized elsewhere and then transported to that tissue. In contrast, tissue specific RNAi knockdown of a SL biosynthesis gene would only decrease SL biosynthesis in that tissue, but it would not prevent accumulation of SL synthesized elsewhere and transported into the tissue. Promoters of differing strengths could be chosen for *AtCXE20* expression to identify and achieve optimal reductions in SL concentrations. Fine-tuning the optimal level of SL or SL signaling might in some cases be more difficult with RNAi knockdowns of SL biosynthesis or signaling genes. It may also be possible to engineer AtCXE20 variants that effectively sequester only some, but not all SL family members, depending on the relative binding affinities of the variant for each SL structure. The straightforward method provided here to obtain functional AtCXE20 protein following expression in *E. coli* could enable such a protein engineering approach.

The catalytic triad and a few other features of α/β hydrolase fold proteins are well-conserved, but overall sequence identities can be quite low (Ollis et al., 1992). Therefore, functions of α/β hydrolase fold proteins are difficult to predict based on amino acid sequence alone. Consistent with this principle, AtCXE20 had almost no sequence identity with known SL receptors. The discovery of the SL-binding function of AtCXE20 would, therefore, have been unlikely if not for the phenotypes observed in *AtCXE20*-overexpressing *Arabidopsis* and maize plants, which so closely resembled known phenotypes of SL-deficient plants and provided rationale for the biochemical experiments with SL. Strong constitutive expression of other members of this protein fold may lead to other gains of function, some of which may be useful, but predicting outcomes ahead of time would be challenging, and elucidating the mechanism may depend on whether informative and diagnostic phenotypes are apparent in the transgenic plants.

In summary, this study identified an agriculturally important role for SL in maize silk growth, provided a new approach for altering SL levels in plants, and gave insights into the gain of new functions in the α/β hydrolase fold protein family.

## Materials and Methods

### Purification and Biochemical Characterization of Recombinant AtCXE20

Recombinant AtCXE20 protein was obtained by expression with the pET28a vector (Novagen) in *E. coli* strain BL21 Gold (DE3; Stratagene). The protein contained a 20-amino acid N-terminal peptide fusion (mgsshhhhhhssglvprgsh) that included a 6-histidine tag to facilitate purification. The *E. coli* cultures were grown at 37°C in 2X YT media to an OD_600nm_ of 0.6. Transgene expression was then induced with 0.5 mM IPTG and the culture was grown an additional 6 h at 20°C. The fusion protein was purified from *E. coli* extracts using cobalt affinity chromatography. The pellet from a 300 ml culture was lysed at room temperature in 10 ml of 50 mM Tris-HCl pH 8, 100 mM NaCl, 0.1% TritonX-100, 0.1 mg/ml lysozyme, and 100 μl of Protease Inhibitor Cocktail Set III (Calbiochem). After 20 min, 5 μl Benzonase Nuclease (Novagen) was added for an additional 10 min to reduce viscosity. The lysate was centrifuged at 4°C for 15 min at 15,000 *g* and the supernatant was then incubated with 4 ml of HisPur Cobalt Resin (Thermo Scientific) for 20 min before pouring into a column and allowing unbound protein to flow through. The column was washed with 20 ml of 50 mM Tris-HCl pH 8, 200 mM NaCl, and 10 mM imidazole, followed by elution with 50 mM Tris-HCl pH 8, 200 mM NaCl, and 60 mM imidazole. Aliquots of the purified protein containing 10% glycerol were quickly frozen in liquid nitrogen and then stored at −80°C. Aliquots were thawed as needed and dialyzed against 50 mM Tris-HCl pH 8, followed by addition of 5 mM TCEP and storage at 4°C. Addition of the TCEP reductant was important, because substantial loss of activity was observed during storage at 4°C in the absence of reductant. Dialyzed protein was quantitated by absorbance at 280 nm, using a value of 1 OD (280 nm) = 0.92 mg/ml, obtained from Vector NTI. Esterase assays with p-nitrophenyl acetate as substrate were done in 50 mM Tris-HCl, pH 8, with 1 μg of protein in an assay volume of 200 μl, using 96-well flat bottom microtiter plates. The increase in absorbance at 405 nm was monitored, and rates of control reactions without enzyme were subtracted to correct for background due to autohydrolysis of substrate. Kinetic parameters, SEs, and Ki values were determined using the enzyme kinetics module of SigmaPlot 12.5.

### Modeling of AtCXE20 3D Structure

The AeCXE1 structure (PDB:2o7v) was selected as modeling template. Based on the target-template alignment, 20 different three-dimensional (3D) models were generated by MODELER 9.15 in Discovery Studio (DS; Accelrys Software Inc., BIOVIA 2016 release) and the model with the lowest discrete optimized protein energy (DOPE) score was used for ligand docking and analysis. The quality of the model was verified with MODELER, which yielded a normalized DOPE score of <−1.66 and showed that all side chains except S166 resided in the allowed area in the Ramachandran plot. Molecular Dynamic simulation of the AtCXE20 homologous model was carried out with GROMACS 5.1.4 (Van Der Spoel et al., 2005) on Linux for 35 nanoseconds. The ligand binding tunnels were calculated with CAVER plugin (Jurcik et al., 2018) in PyMOL, and all the graphs are prepared with PyMOL (Schrodinger, LLC).

### Screening Enhancer-Tagged *Arabidopsis* Library for Drought Tolerant Lines

Plants from an *Arabidopsis thaliana* (ecotype Columbia) enhancer-tagged library were grown in flats until 15–20 days after planting, and then watering was stopped until bolting stage approximately 2 weeks later. Green leaf area was monitored during the drought treatment by using LemnaTec HTSBonitUV software as described previously (Shi et al., 2015). Watering was then resumed for the duration of the experiment to allow harvest of viable seeds.

### Generation of Maize Transgenic Events

*Agrobacterium tumefaciens*-mediated transformation of maize inbred line PHR03 was carried out with the *AtCXE20* transformation cassette described in Figure 2A that used the maize ubiquitin promoter including the first intron (Christensen et al., 1992) and the sorghum gamma kaffirin terminator to control *AtCXE20* expression. The selectable marker was maize optimized phosphinothricin acetyltransferase (MOPAT) which provided resistance to the herbicide glufosinate. Hybrid seed for the 2014 field studies was produced by crossing the transformed PHR03 inbred events with inbred PH12SG. Subsequently, a 2nd maize inbred line PH184C was transformed with a similar cassette as Figure 2A but with no DSRED color marker. Hybrid seed for the 2016 field studies was produced by crossing the PH184C transgenic events with inbred line PH1V69. Quantitative PCR was used to determine that each transgenic event advanced for field studies had a single copy each of the *AtCXE20* transgene and the MOPAT selectable marker, except for PHR03 event E3 which had two copies of the *AtCXE20* transgene.

### Hybrid Maize Field Plot Experiments to Determine Tiller Numbers, Transgene Expression, and Quantity of AtCXE20 Protein

A randomized complete block design was used with two-row plots and four field blocks. Row length was 4.4 m, and row width was 76.2 cm. Each block included one plot for each transgenic event and two plots for null. The maize hybrid was PHR03 × PH12SG. The number of tillers/plant was determined at growth stage V12, using all plants in the plot except for the end plants of each row.

Leaf samples for transgene expression analysis were obtained at growth stage R2 and were immediately frozen in liquid nitrogen. Total RNA was extracted with RNeasy reagents (Qiagen), and cDNA was synthesized with a mixture of oligo(dT) and random hexamer primers using Applied Biosystems HCAP Reverse Transcription kit (Life Technologies). Quantitative RT-PCR (qRT-PCR) was performed with a TaqMan hydrolysis probe using the TaqMan Universal Master Mix (Life Technologies). Relative quantification values were determined using the difference in Ct between the target gene and the internal control, maize eIF4-γ.

The quantity of AtCXE20 protein in maize leaves and roots was determined by liquid chromatography coupled with tandem mass spectrometry (LC-MS/MS). Leaf samples from plots for three transgenic events (E1, E2, and E3) and null were obtained at growth stage V9 by pooling one leaf punch per plant for 10 plants per plot, and quick freezing in liquid nitrogen. Primary root samples from 1-week old seedlings grown in the dark in moist paper rolls were obtained from transgenic events E2 and E3, and null. The samples were ground in liquid nitrogen, and the frozen powder was lyophilized. The protein from 10 mg of lyophilized powder per sample was extracted with 600 μl PBST buffer, normalized to a concentration of 0.8 mg/ml protein, reduced with dithiothreitol, alkylated with iodoacetamide, and digested for 18 h overnight at 37°C with trypsin (1:25 trypsin/protein ratio). Formic acid was added to stop the digestion and to assist in ionization followed by the addition of isotopically labeled internal standard. Digested extract (10 μl) was injected onto a 2.1 mm x 50 mm ultra-performance liquid chromatography (UPLC) column from Waters (BEH C18 1.7 μm) set at 60°C. Mobile phases were 0.1% formic acid in water (MPA) and 0.1% formic acid in acetonitrile (MPB). Separation was achieved with a 1.5 min linear gradient of 8–30% MPB. The LC-MS/MS system included a SCIEX 5500 Q-TRAP with a Turbo ion-spray source. Recombinant AtCXE20 protein was spiked into null matrix to produce a standard curve with a linear dynamic range of 50X. The AtCXE20 tryptic peptide DLNAIVVSPSYR was used to quantitate AtCXE20 protein abundance. Targeted analyses of MRM 667.3/807.4 for DLNAIVVSPSYR and MRM 670.3/813.4 for DLNAIV*VSPSYR internal standard were completed using Analyst software (SCIEX) for data acquisition and quantification. The parent ions for the analyte and internal standard are noted as follows: DLNAIVVSPSYR, 667.3 and DLNAIV*VSPSYR, 670.3. The heavy-labeled Valine (noted as V*) was labeled with carbon 13 (C13) and nitrogen 15 (N15), which increases the mass of valine by 6 Daltons (Da). Because the internal standard is a +2 charge state, the addition of the heavy-labeled valine (V-C13,N15) increases the parent ion mass by 3 Da (3 Da difference between DLNAIVVSPSYR, 667.3 and DLNAIV*VSPSYR, 670.3). The product ions for the analyte and internal standard are noted as follows: DLNAIVVSPSYR, 807.4 and DLNAIV*VSPSYR, 813.4. The y7 product ion was measured and corresponds to the following analyte and internal standard fragment sequences, VVSPSYR and V*VSPSYR. Because the product ion is a +1 charge state, the addition of the heavy-labeled valine (V-C13,N15) increases the product ion mass by 6 Da (6 Da difference between DLNAIVVSPSYR, 807.4 and DLNAIV*VSPSYR, 813.4).

### Hybrid Maize Field Pot Experiments for Phenotypic Analysis in Drought Stressed and Water-Replete Conditions

Hybrid maize plants were grown outdoors in 2014 and 2016 in Johnston, Iowa in 10-L pots supported by wooden racks, with one plant per pot. The hybrids used were PHR03 × PH12SG for 2014 and PH184C × PH1V69 for 2016. The pots (TreepotTP818, Stuewe & Sons) were filled with an equal weight of 4,500 g potting soil (Fafard 3B mix). The pots were rectangular with dimensions at the top of 19.1 cm x 45.7 cm (7.5''x18''). Each wooden support rack was 250 cm long, 60 cm wide, and 55 cm high and held two rows of 12 pots each. Pots were irrigated with Peters Excel fertilizer (Everris NA Inc.) adjusted to provide 100 ppm N. The fertilizer contained 15% N, 5% P_2_O_5_, 15% K_2_O, 5% Ca, 2% Mg, 0.0187% B, 0.0187% Cu, 0.075% Fe, 0.0375% Mn, 0.0075% Mo, and 0.0375% Zn. Weather information and drought treatment timing are presented in Supplementary Table 1. For the water-replete treatment, pots were irrigated daily throughout the growing season. For the drought treatment, a series of drought stresses were sequentially imposed by withholding irrigation and letting pots drain and dry out until pronounced wilting was observed, corresponding to a leaf relative water content of 60–65%, followed by resuming irrigation until the next stress treatment was started. Seven and four stress treatments were imposed in 2014 and 2016, respectively. Fifteen and 18 replications for each environmental treatment were used in 2014 and 2016, respectively. There were six pots per replication comprising three transgenic events and three controls. Segregating event nulls were used as controls in 2014, and wild type plants were used as controls in 2016. Border plants were included on each side and each end of the experiment. Plant height was measured to the collar of the uppermost fully expanded leaf, and tillers per plant were counted at growth stages V6 and V8 in 2014 and 2016, respectively. Leaf flavonols and chlorophyll were determined on the leaf above the ear leaf using a Dualex Scientific instrument (Force-A) and measuring midway between the midrib and the leaf edge, and midway between the leaf tip and the stalk. The ASI for a plant was calculated as the day of first silk emergence for the plant minus the day of first pollen shed for the same plant. At physiological maturity, plants were cut at soil level, and ears were removed and shelled. The plant materials were dried in burlap bags at 70°C until weight stopped decreasing, prior to determining dry weight of total aboveground biomass and seed. Harvest index for a plant was calculated as seed dry weight divided by total dry weight.

### Statistical Methods

For the field pot study, the experimental design was a multi-rack split-plot design. Events were randomized in main plots, and event positive and negative pairs were randomized in sub plots to ensure that throughout the experiment, each transgenic plant was adjacent to an appropriate control plant. Each rack contained multiple replications (usually four). A linear mixed model was applied to model traits for each environmental treatment separately. Data for trait (*Y_ijnms_*) of rack ${\left(L\right)}_{i}$, replication (*R*)*_j_*, event (*E*)*_n_*, genotype (*G*)*_m_*, and plant *s*, were modeled as a function of an overall mean *μ*, factors for rack, rack by replication, event, rack by event, rack by replication by event, genotype, genotype by event, rack by genotype, and a residual within each rack ${\left(\varepsilon /L\right)}_{ijns}$. The model can be specified as:

$$\begin{array}{l} Y_{ijnms}=\mu +L_{i}\;+\;{\left(L\times R\;\right)}_{ij}\;+E_{n}+\;\;{\left(L\times E\right)}_{in}\;\;+\;{\left(L\times R\times E\right)}_{ijn}\;\;\;+\\ \;\;\;\;\;\;\;\;\;\;\;\;\;\;\;\;\;\;\;\;\;\;\;\;\;G_{m}+{\left(E\times G\right)}_{nm}+\;{\left(L\times G\right)}_{im}\;+{\left(\varepsilon /L\right)}_{ijnms} \end{array}$$

where event and genotype were treated as fixed effect, and all the other effects except the residual were treated as independent normally distributed random variables with means of zero. *T*-tests using SEs from the model were conducted to compare treatment effects. All data analysis and comparisons were made in ASReml 3.0 (VSN International, Hemel Hempstead, United Kingdom, 2009).

For the field plot study, the experimental design was a randomized complete block design with events randomized within blocks (replications). A linear mixed model was used. Data for trait (*Y_ins_*) of replication (*R*)*_i_*, event (*E*)*_n_*, and plant *s*, were modeled as a function of an overall mean *μ*, factors for replication, event, and a residual ɛ*_ins_*. The model can be specified as:

$$Y_{ins}=\mu +\;R_{i}\;\;+E_{n}+\varepsilon_{ins}$$

where event was treated as fixed effect, and all the other effects were treated as independent normally distributed random variables with means of zero. *T*-tests using SEs from the model were conducted to compare treatment effects. All data analysis and comparisons were made in ASReml 3.0 (VSN International, Hemel Hempstead, United Kingdom, 2009).

For the lab studies, the branching, ACC sensitivity, esterase activity, fluorescence, and silk growth, experiments were statistically analyzed by two sample *t*-tests, assuming equal variances (Microsoft Excel).

### Determination of Maize Silk Growth *in vitro* With or Without Exogenous GR24

Transgenic and segregating null plants of maize hybrid PHR03 × PH12SG event E3 were grown in a greenhouse in 20-L pots. Supplemental lighting at an intensity of 800 μmol photons/m^2^/s was used to provide a photoperiod of 16 h light/8 h dark. Daytime and nighttime temperatures were kept at 26 and 20°C, respectively, and relative humidity was kept at 60%. At 1 day after silking, ears were removed from greenhouse-grown plants and placed in a bucket of water for transport to the lab. In a sterile hood, husks were removed and 3-cm lengths of silks were excised from the area shown in Figure 5A. Half of the silks were put in 50 ml polycarbonate tubes containing 20 ml of 1x MS basal salts, 1% sucrose, 50 μg/ml kanamycin sulfate, and 10 μM GR24 (10 μl of a 20 mM stock in DMSO). The other half were placed in a tube containing the identical volume and composition except that 10 μl DMSO without GR24 were included. Three ears each for transgenic and null were sampled. The tubes were incubated at room temperature (approximately 22°C) on a revolving mixer for 48 h. Ten silks per tube were then measured manually, and growth was calculated as total length minus the 3-cm starting length.

### Reagents

The rac-GR24 (product number CX23880) was purchased from Chiralix (The Netherlands). The 5-deoxystrigol [the (+) isomer, product number 0257123] and karrikin 2 (product number 0256823) were purchased from OlchemIm Ltd (the Czech Republic). Other hormones and p-nitrophenyl acetate were purchased from Sigma-Aldrich (United States).

## Data Availability Statement

The original contributions presented in the study are included in the article/Supplementary Material, further inquiries can be directed to the corresponding author.

## Author Contributions

Research designed by CL, RW, KR, JT, QX, LL, and JH. Research performed by CL, JT, QX, PV, ZH, MO, and KR. Data analyzed by LL, QX, CL, and KR. Project supervised by RW and JH. Manuscript written by KR with input from all authors. All authors contributed to the article and approved the submitted version.

### Conflict of Interest

All authors are or were employees of Corteva Agriscience or its parent companies when they carried out this research. Corteva Agriscience is a for-profit agricultural technology company. Patent applications related to this work have been filed.

## References

[ref1] Akiyama K. Matsuzaki K.-I. Hayashi H. (2005). Plant sequiterpenes induce hyphal branching in arbuscular mycorrhizal fungi. Nature 435, 824–827. 10.1038/nature03608, PMID: 15944706

[ref2] Al-Babili S. Bouwmeester H. J. (2015). Strigolactones, a novel carotenoid-derived plant hormone. Annu. Rev. Plant Biol. 66, 161–186. 10.1146/annurev-arplant-043014-114759, PMID: 25621512

[ref3] Albe K. R. Butler M. H. Wright B. E. (1990). Cellular concentrations of enzymes and their substrates. J. Theor. Biol. 143, 163–195. 10.1016/S0022-5193(05)80266-8, PMID: 2200929

[ref4] Alder A. Jamil M. Marzorati M. Bruno M. Vermathen M. Bigler P.. (2012). The path from β-carotene to carlactone, a strigolactone-like plant hormone. Science 335, 1348–1351. 10.1126/science.1218094, PMID: 22422982

[ref5] Awad A. A. Sato D. Kusumoto D. Kamioka H. Takeuchi Y. Yoneyama K. (2006). Characterization of strigolactones, germination stimulants for the root parasitic plants Striga and Orobanche, produced by maize, millet, and sorghum. Plant Growth Regul. 48, 221–227. 10.1007/s10725-006-0009-3

[ref6] Besserer A. Puech-Pages V. Kiefer P. Gomez-Roldan V. Jauneau A. Roy S.. (2006). Strigolactones stimulate arbuscular mycorrhizal fungi by activating mitochondria. PLoS Biol. 4:e226. 10.1371/journal.pbio.0040226 16787107 PMC1481526

[ref7] Bolanos J. Edmeades G. O. (1996). The importance of anthesis-silking interval in breeding for drought tolerance in tropical maize. Field Crop Res. 48, 65–80. 10.1016/0378-4290(96)00036-6

[ref8] Boyer J. S. (1982). Plant productivity and environment. Science 218, 443–447. 10.1126/science.218.4571.443, PMID: 17808529

[ref9] Bu Q. Lv T. Shen H. Luong P. Wang J. Wang Z.. (2014). Regulation of drought tolerance by the f-box protein MAX2 in *Arabidopsis*. Plant Physiol. 164, 424–439. 10.1104/pp.113.226837, PMID: 24198318 PMC3875819

[ref10] Campos H. Cooper M. Habben J. E. Edmeades G. O. Schussler J. R. (2004). Improving drought tolerance in maize: a view from industry. Field Crop Res. 90, 19–34. 10.1016/j.fcr.2004.07.003

[ref11] Carr P. D. Ollis D. L. (2009). Alpha/beta hydrolase fold: an update. Protein Pept. Lett. 16, 1137–1148. 10.2174/092986609789071298, PMID: 19508187

[ref12] Charnikhova T. V. Gaus K. Lumbroso A. Sanders M. Vincken J.-P. Mesmaeker A. D.. (2017). Zealactones. Novel natural strigolactones from maize. Phytochemistry 137, 123–131. 10.1016/j.phytochem.2017.02.010, PMID: 28215609

[ref13] Christensen A. H. Sharrock R. A. Quail P. H. (1992). Maize polyubiquitin genes: structure, thermal perturbation of expression and transcript splicing, and promoter activity following transfer to protoplasts by electroporation. Plant Mol. Biol. 18, 675–689. 10.1007/BF00020010, PMID: 1313711

[ref14] Conn C. E. Bythell-Douglas R. Neumann D. Yoshida S. Whittington B. Westwood J. H.. (2015). Convergent evolution of strigolactone perception enabled host detection in parasitic plants. Science 349, 540–543. 10.1126/science.aab1140, PMID: 26228149

[ref15] Cummins I. Landrum M. Steel P. G. Edwards R. (2007). Structure activity studies with xenobiotic substrates using carboxylesterases isolated from *Arabidopsis thaliana*. Phytochemistry 68, 811–818. 10.1016/j.phytochem.2006.12.014, PMID: 17270225

[ref16] Danilevskaya O. N. GongXin Y. Meng X. Xu J. Stephenson E. Estrada S.. (2019). Developmental and transcriptional responses of maize to drought stress under field conditions. Plant Direct 3, 1–20. 10.1002/pld3.129 PMC6589525 31245774

[ref17] de Saint Germain A. Clave G. Badet-Denisot M.-A. Pillot J.-P. Cornu D. Le Car J.-P.. (2016). An histidine covalent receptor and butanolide complex mediates strigolactone perception. Nat. Chem. Biol. 12, 787–794. 10.1038/nchembio.2147, PMID: 27479744 PMC5030144

[ref18] Devonshire A. L. Moores G. D. (1982). A carboxylesterase with broad substrate specificity cause organophosphorus, carbamate and pyrethroid resistance in peach-potato aphids (*Myzus persicae*). Pestic. Biochem. Physiol. 18, 235–246. 10.1016/0048-3575(82)90110-9

[ref19] Emanuelsson O. Nielsen H. Brunak S. von Heijne G. (2000). Predicting subcellular localization of proteins based on their N-terminal amino acid sequence. J. Mol. Biol. 300, 1005–1016. 10.1006/jmbi.2000.3903, PMID: 10891285

[ref20] Field L. M. Devonshire A. L. Forde B. G. (1988). Molecular evidence that insecticide resistance in peach-potato aphids (*Myzus persicae* Sulz.) results from amplification of an esterase gene. Biochem. J. 251, 309–312. 10.1042/bj2510309, PMID: 3390158 PMC1148999

[ref21] Fischer K. S. Edmeades G. O. Johnson E. C. (1989). Selection for the improvement of maize yield under moisture-defiicits. Field Crop Res. 22, 227–243. 10.1016/0378-4290(89)90094-4

[ref22] Flokova K. Shimels M. Jimenez B. A. Bardaro N. Strnad M. Novak O.. (2020). An improved strategy to analyse strigolactones in complex sample matrices using UHPLC-MS/MS. Plant Methods 16:125. 10.1186/s13007-020-00669-3 32963580 PMC7499983

[ref23] Fuad-Hassan A. Tardieu F. Turc O. (2008). Drought-induced changes in anthesis-silking interval are related to silk expansion: a spatio-temporal growth analysis in maize plants subjected to soil water deficit. Plant Cell Environ. 31, 1349–1360. 10.1111/j.1365-3040.2008.01839.x, PMID: 18518916

[ref24] Gomez-Roldan V. Fermas S. Brewer P. B. Puech-Pages V. Dun E. A. Pillot J. P.. (2008). Strigolactone inhibition of shoot branching. Nature 455, 189–194. 10.1038/nature07271, PMID: 18690209

[ref25] Guan J. C. Koch K. E. Suzuki M. Wu S. Latshaw S. Petruff T.. (2012). Diverse roles of strigolactone signaling in maize architecture and the uncoupling of a branching-specific subnetwork. Plant Physiol. 160, 1303–1317. 10.1104/pp.112.204503, PMID: 22961131 PMC3490586

[ref26] Ha C. V. Leyva-Gonzalez M. A. Osakabe Y. Tran U. T. Nishiyama R. Watanabe Y.. (2014). Positive regulatory role of strigolactone in plant responses to drought and salt stress. Proc. Natl. Acad. Sci. U. S. A. 111, 851–856. 10.1073/pnas.1322135111 24379380 PMC3896162

[ref27] Habben J. E. Schussler J. R. (2017). “Effects of Drought Stress on Maize Kernel Set,” in Maize Kernel Development. ed. Larkins B. A. (Boston, MA: CAB International), 217–229.

[ref28] Haider I. Andreo-Jimenez B. Bruno M. Bimbo A. Flokova K. Abuauf H.. (2018). The interaction of strigolactones with abscisic acid during drought response in rice. J. Exp. Bot. 69, 2403–2414. 10.1093/jxb/ery089, PMID: 29538660

[ref29] Horton P. Park K.-J. Obayashi T. Fujita N. Harada H. Adams-Collier C. J.. (2007). WoLF PSORT: protein localization predictor. Nucleic Acids Res. 35, W585–W587. 10.1093/nar/gkm259, PMID: 17517783 PMC1933216

[ref30] Ileperuma N. R. Marshall S. D. G. Squire C. J. Baker H. M. Oakeshott J. G. Russell R. J.. (2007). High-resolution crystal structure of plant carboxylesterase AeCXE1, from *Actinidia eriantha*, and its complex with a high-affinity inhibitor paraoxon. Biochemistry 46, 1851–1859. 10.1021/bi062046w, PMID: 17256879

[ref31] Jiang L. Liu X. Xiong G. Liu H. Chen F. Wang L.. (2013). DWARF 53 acts as a repressor of strigolactone signaling in rice. Nature 504, 401–405. 10.1038/nature12870, PMID: 24336200 PMC5802366

[ref32] Jurcik A. Bednar D. Byska J. Marques S. M. Furmanova K. Daniel L.. (2018). CAVER analyst 2.0: analysis and visualization of channels and tunnels in protein structures and molecular dynamics trajectories. Bioinformatics 34, 3586–3588. 10.1093/bioinformatics/bty386, PMID: 29741570 PMC6184705

[ref33] Kapulnik Y. Delaux P.-M. Resnick N. Mayzlish-Gati E. Wininger S. Bhattacharya C.. (2011). Strigolactones affect lateral root formation and root-hair elongation in *Arabidopsis*. Planta 233, 209–216. 10.1007/s00425-010-1310-y, PMID: 21080198

[ref34] Ko M. K. Jeon W. B. Kim K. S. Lee H. H. Seo H. H. Kim Y. S.. (2005). A colletotrichum gloeosporioides-induced esterase gene of nonclimacteric pepper (*Capsicum annuum*) fruit during ripening plays a role in resistance against fungal infection. Plant Mol. Biol. 58, 529–541. 10.1007/s11103-005-7088-9, PMID: 16021337

[ref35] Lee S. Hwang S. Seo Y. W. Jeon W. B. Oh B.-J. (2013). Molecular characterization of the *AtCXE8* gene, which promotes resistance to *Botrytis cinereal* infection. Plant Biotechnol. Rep. 7, 109–119. 10.1007/s11816-012-0253-0

[ref36] Liu J. He H. Vitali M. Visentin I. Charnikhova T. Haider I.. (2015). Osmotic stress represses strigolactone biosynthesis in *Lotus japonicas* roots: exploring interaction between strigolactones and ABA under abiotic stress. Planta 241, 1435–1451. 10.1007/s00425-015-2266-8, PMID: 25716094

[ref37] Marchot P. Chatonnet A. (2012). Enzymatic activity and protein interactions in alpha/beta hydrolase fold proteins: moonlighting versus promiscuity. Protein Pept. Lett. 19, 132–143. 10.2174/092986612799080284, PMID: 21933125

[ref38] Marshall S. D. G. Putterill J. J. Plummer K. M. Newcomb R. D. (2003). The carboxylesterase gene family from *Arabidopsis thaliana*. J. Mol. Evol. 57, 487–500. 10.1007/s00239-003-2492-8, PMID: 14738307

[ref39] Needleman S. B. Wunsch C. D. (1970). A general method applicable to the search for similarities in the amino acid sequence of two proteins. J. Mol. Biol. 48, 443–453. 10.1016/0022-2836(70)90057-4, PMID: 5420325

[ref40] Ollis D. L. Cheah E. Cygler M. Dijkstra B. Frolow F. Franken S. M.. (1992). The α/β hydrolase fold. Protein Eng. 5, 197–211. 10.1093/protein/5.3.197, PMID: 1409539

[ref41] Oury V. Caldeira C. F. Prodhomme D. Picho J.-P. Gibon Y. Tardieu F.. (2016a). Is change in ovary carbon status a cause or a consequence of maize ovary abortion in water deficit during flowering? Plant Physiol. 171, 997–1008. 10.1104/pp.15.01130 27208256 PMC4902574

[ref42] Oury V. Tardieu F. Turc O. (2016b). Ovary apical abortion under water deficit is caused by changes in sequential development of ovaries and in silk growth rate in maize. Plant Physiol. 171, 986–996. 10.1104/pp.15.00268 26598464 PMC4902573

[ref43] Rome L. H. Lands W. E. M. (1975). Structural requirements for time-dependent inhibition of prostaglandin biosynthesis by anti-inflammatory drugs. Proc. Natl. Acad. Sci. U. S. A. 72, 4863–4865. 10.1073/pnas.72.12.4863 1061075 PMC388832

[ref44] Seto Y. Yasui R. Kameoka H. Tamiru M. Cao M. Terauchi R.. (2019). Strigolactone perception and deactivation by a hydrolase receptor DWARF 14. Nat. Commun. 10:191. 10.1038/s41467-018-08124-7 30643123 PMC6331613

[ref45] Shi J. Habben J. E. Archibald R. L. Drummond B. J. Chamberlin M. A. Williams R. W.. (2015). Overexpression of Argos genes modifies plant sensitivity to ethylene, leading to improved drought tolerance in both *Arabidopsis* and maize. Plant Physiol. 169, 266–282. 10.1104/pp.15.00780, PMID: 26220950 PMC4577415

[ref46] Toh S. Holbrook-Smith D. Stogios P. J. Onopriyenko O. Lumba S. Tsuchiya Y.. (2015). Structure-function analysis identifies highly sensitive strigolactone receptors in Striga. Science 350, 203–207. 10.1126/science.aac9476, PMID: 26450211

[ref47] Tsuchiya Y. Yoshimura M. Sato Y. Kuwata K. Toh S. Holbrook-Smith D.. (2015). Probing strigolactone receptors in *Striga hermonthica* with fluorescence. Science 349, 864–868. 10.1126/science.aab3831, PMID: 26293962

[ref48] Ueda H. Kusaba K. (2015). Strigolactone regulates leaf senescence in concert with ethylene in *Arabidopsis*. Plant Physiol. 169, 138–147. 10.1104/pp.15.00325, PMID: 25979917 PMC4577378

[ref49] Ueguchi-Tanaka M. Ashikari M. Nakajima M. Itoh H. Katoh E. Kobayashi M.. (2005). *Gibberellin insensitive Dwarf1* encodes a soluble receptor for gibberellin. Nature 437, 693–698. 10.1038/nature04028, PMID: 16193045

[ref50] Umehara M. Hanada A. Yoshida S. Akiyama K. Arite T. Takeda-Kamiya N.. (2008). Inhibition of shoot branching by new terpenoid plant hormones. Nature 455, 195–200. 10.1038/nature07272, PMID: 18690207

[ref51] Van Der Spoel D. Lindahl E. Hess B. Groenhof G. Mark A. E. Berendsen H. J. C. (2005). GROMACS: fast, flexible, and free. J. Comput. Chem. 26, 1701–1718. 10.1002/jcc.20291 16211538

[ref52] Walton A. Stes E. Goeminne G. Braem L. Vuylsteke M. Matthys C.. (2016). The response of the root proteome to the synthetic strigolactone GR24 in *Arabidopsis*. Mol. Cell. Proteomics 15, 2744–2755. 10.1074/mcp.M115.050062, PMID: 27317401 PMC4974348

[ref53] Wang Y. Shang L. Yu H. Zeng L. Hu J. Ni S.. (2020). A strigolactone biosynthesis gene contributed to the green revolution in rice. Mol. Plant 13, 923–932. 10.1016/j.molp.2020.03.009, PMID: 32222483

[ref54] Wang B. Smith S. M. Li J. (2018). Genetic regulation of shoot architecture. Annu. Rev. Plant Biol. 69, 437–468. 10.1146/annurev-arplant-042817-040422, PMID: 29553800

[ref55] Weigel D. Ahn J. H. Blazquez M. A. Borevitz J. O. Christensen S. K. Fankhauser C.. (2000). Activation tagging in *Arabidopsis*. Plant Physiol. 122, 1003–1013. 10.1104/pp.122.4.1003, PMID: 10759496 PMC1539247

[ref56] Wu J. Lawit S. J. Weers B. Sun J. Monger N. Van Hemert J.. (2019). Overexpression of zmm28 increases maize grain yield in the field. Proc. Natl. Acad. Sci. U. S. A. 116, 23850–23858. 10.1073/pnas.1902593116, PMID: 31685622 PMC6876154

[ref57] Yoneyama K. Arakawa R. Ishimoto K. Kim H. I. Kisugi T. Xie X.. (2015). Difference in Striga-suceptibility is reflected in strigolactone secretion profile, but not in compatibility and host preference in arbuscular mycorrhizal symbiosis in two maize cultivars. New Phytol. 206, 983–989. 10.1111/nph.13375, PMID: 25754513

[ref500] Zhou F. Lin Q. Lihong Z. Ren Y. Zhou K. Shabok N.. (2013). D14-SCFD3-dependent degradation of D53 regulates strigolactone signalling. Nature 504, 406–410. 10.1038/nature12878 24336215 PMC4096652

