# Supplementary material for: Arabidopsis Carboxylesterase 20 Binds Strigolactone and Increases Branches and Tillers When Ectopically Expressed in Arabidopsis and Maize
Source: Front Plant Sci. 2021 Apr 27;12:639401. doi: 10.3389/fpls.2021.639401 (PMC8110907; doi:10.3389/fpls.2021.639401)
Supplement: Supplementary file 1 [file Data_Sheet_1.PDF]

Supplemental Table 1. Timing of drought stress treatments for 2014 and 2016 field pot experiments and rains occurring during treatment period.

**Date**	**Description**
5/21/14	Planting date
6/19/14	tillers counted
6/30/14	Rain (7.26 cm); started DRT treatment 1
7/1/14	V10 plant height measured
7/5/14	Rain (4.72 cm)
7/11/14	Irrigated; started DRT treatment 2
7/12/14	Rain (0.66 cm)
7/16/14	V16 plant height measured
7/18/14	Irrigated; started DRT treatment 3
7/23/14	Irrigated; started DRT treatment 4
7/30/14	Irrigated; started DRT treatment 5
8/5/14	Irrigated; started DRT treatment 6
8/6/14	Rain (5.51 cm)
8/7/14	Rain (0.53 cm)
8/11/14	Yellow leaves/plant counted
8/14/14	Irrigated; started DRT treatment 7
8/15/14	Rain (0.86 cm)
8/20/14	Irrigated; maintained irrigation through rest of season
5/26/16	Planting date
6/23/16	Started DRT treatment 1; tillers counted
6/26/16	Rain (0.79 cm)
6/27/16	V9 plant height measured
6/29/16	Rain (0.36 cm)
6/30/16	Irrigated; rain (1.09 cm); started DRT treatment 2
7/6/16	Rain (1.14 cm)
7/7/16	Irrigated; rain (2.39 cm); started DRT treatment 3
7/10/16	Rain (0.25 cm)
7/11/16	Rain (2.39)
7/12/16	Rain (1.09 cm)
7/13/16	Irrigated; rain (0.53 cm); started DRT treatment 4
7/17/16	Rain (1.57 cm)
7/18/16	Rain (4.75 cm)
7/19/16	Irrigated; rain (4.67 cm); maintained watering through rest of season
7/25/16	R2 plant height measured

Supplemental Table 2. Analysis of field pot study by event. Trait abbreviations are as in Table 1.

			Water-replete					Drought-stressed				
Year	Trait	Event	Trans	Ctrl	Diff	*p* value	signif	Trans	Ctrl	Diff	*p* value	signif
2014	Tillers/pl	E1	1.26	0.972	0.285	0.101	+	1.18	0.811	0.367	0.0374	*
2014	Tillers/pl	E2	1.37	0.972	0.4	0.0207	*	1.34	0.611	0.733	4.33E-05	***
2014	Tillers/pl	E3	1.36	0.938	0.422	0.0156	*	1.28	0.744	0.533	0.00264	**
2014	Pl ht V10 (cm)	E1	88.9	91.0	-2.07	0.0563	+	92.2	93.4	-1.21	0.250	NS
2014	Pl ht V10 (cm)	E2	87.8	91.1	-3.25	0.00270	**	91.4	92.3	-0.953	0.377	NS
2014	Pl ht V10 (cm)	E3	82.6	89.1	-6.46	1.18E-08	***	88.5	92.8	-4.30	7.42E-05	***
2014	Pl ht V16 (cm)	E1	190	194	-3.20	0.0267	*	173	174	-0.745	0.606	NS
2014	Pl ht V16 (cm)	E2	188	192	-4.20	0.00337	**	173	173	0.0701	0.961	NS
2014	Pl ht V16 (cm)	E3	180	189	-8.86	5.97E-09	***	172	174	-1.97	0.169	NS
2014	Flavonols	E1	0.826	0.883	-0.0558	0.346	NS	0.735	0.814	-0.0794	0.105	+
2014	Flavonols	E2	0.844	0.876	-0.0318	0.590	NS	0.716	0.858	-0.142	0.00385	**
2014	Flavonols	E3	0.799	0.894	-0.0949	0.110	NS	0.779	0.719	0.0604	0.218	NS
2014	Chlor (µg/cm2)	E1	45.1	44.7	0.404	0.788	NS	41.4	42.1	-0.696	0.559	NS
2014	Chlor (µg/cm2)	E2	43.9	44.6	-0.745	0.620	NS	41.7	41.0	0.648	0.584	NS
2014	Chlor (µg/cm2)	E3	43.2	44.8	-1.63	0.272	NS	41.8	41.8	0.00891	0.994	NS
2014	Shed (DAP)	E1	61.5	61.4	0.0311	0.838	NS	61.8	62.0	-0.167	0.579	NS
2014	Shed (DAP)	E2	61.6	61.5	0.137	0.369	NS	62	62.4	-0.467	0.121	NS
2014	Shed (DAP)	E3	62.0	61.5	0.542	6.64E-04	***	62.6	61.8	0.800	0.00835	**
2014	Silk (DAP)	E1	62.5	62.4	0.0552	0.745	NS	65.1	65.7	-0.550	0.240	NS
2014	Silk (DAP)	E2	62.7	62.5	0.258	0.129	NS	65.2	65.3	-0.0952	0.834	NS
2014	Silk (DAP)	E3	62.9	62.5	0.395	0.0242	*	65.0	66.3	-1.28	0.0104	*
2014	ASI (days)	E1	0.999	0.968	0.0303	0.880	NS	3.38	3.88	-0.500	0.341	NS
2014	ASI (days)	E2	1.13	1.00	0.130	0.520	NS	3.51	2.89	0.619	0.228	NS
2014	ASI (days)	E3	0.894	1.04	-0.145	0.483	NS	2.48	4.64	-2.17	1.48E-04	***
2014	YL/pl R4	E1	ND	ND	ND	ND	ND	11.0	11.8	-0.771	0.00606	**
2014	YL/pl R4	E2	ND	ND	ND	ND	ND	10.8	10.9	-0.0383	0.891	NS
2014	YL/pl R4	E3	ND	ND	ND	ND	ND	10.4	10.9	-0.509	0.0727	+
2014	Tot DW (g/pl)	E1	411	414	-2.24	0.916	NS	218	205	13.7	0.258	NS
2014	Tot DW (g/pl)	E2	405	374	30.9	0.152	NS	220	217	3.00	0.804	NS
2014	Tot DW (g/pl)	E3	420	394	26.2	0.203	NS	213	209	3.88	0.768	NS
2014	Seed DW (g/pl)	E1	191	199	-7.44	0.439	NS	95.7	80.0	15.7	0.151	NS
2014	Seed DW (g/pl)	E2	185	182	3.08	0.753	NS	93.7	84.6	8.78	0.393	NS
2014	Seed DW (g/pl)	E3	202	190	11.6	0.234	NS	84.9	62.9	22.0	0.0492	*
2014	Seed no/pl	E1	623	672	-49.4	0.0942	+	374	283	91.0	0.0428	*
2014	Seed no/pl	E2	596	606	-9.41	0.752	NS	341	325	16.5	0.696	NS
2014	Seed no/pl	E3	650	615	35.2	0.238	NS	315	229	86.3	0.0587	+
2014	100 seed wt (g)	E1	30.6	29.6	1.05	0.254	NS	26.1	27.6	-1.50	0.156	NS
2014	100 seed wt (g)	E2	31.2	30.2	1.05	0.263	NS	28.1	26.9	1.19	0.228	NS
2014	100 seed wt (g)	E3	31.0	30.8	0.197	0.834	NS	27.1	26.8	0.320	0.769	NS
2014	Harvest index	E1	0.479	0.495	-0.0156	0.258	NS	0.39	0.315	0.0755	0.148	NS

2014	Harvest index	E2	0.466	0.496	-0.029	0.0356	*	0.362	0.366	-0.004	0.938	NS
2014	Harvest index	E3	0.501	0.484	0.0171	0.201	NS	0.336	0.209	0.128	0.0254	*
2016	Tillers/pl	E9	2.46	2.46	0	0.995	NS	2.48	2.56	-0.0726	0.678	NS
2016	Tillers/pl	E10	2.40	2.40	0	0.994	NS	2.56	2.37	0.188	0.271	NS
2016	Tillers/pl	E11	2.47	2.47	0	0.995	NS	2.56	2.30	0.261	0.140	NS
2016	Pl ht V9 (cm)	E9	64.5	65.0	-0.489	0.574	NS	63.2	63.3	-0.101	0.927	NS
2016	Pl ht V9 (cm)	E10	62.5	65.5	-2.98	0.00273	**	61.9	65.6	-3.68	0.00148	**
2016	Pl ht V9 (cm)	E11	62.9	66.1	-3.14	3.44E-04	***	63.2	65.2	-2.06	0.0606	+
2016	Pl ht R2 (cm)	E9	245	247	-2.73	0.138	NS	232	241	-9.27	4.10E-05	***
2016	Pl ht R2 (cm)	E10	245	246	-1.26	0.603	NS	229	240	-10.2	2.90E-05	***
2016	Pl ht R2 (cm)	E11	239	247	-7.92	7.62E-05	***	239	243	-3.34	0.130	NS
2016	Flavonols	E9	0.889	1.06	-0.171	0.00348	**	0.849	1.02	-0.169	6.05E-04	***
2016	Flavonols	E10	0.897	1.03	-0.135	0.0174	*	0.913	1.05	-0.138	0.00502	**
2016	Flavonols	E11	0.973	0.970	0.00290	0.956	NS	0.869	0.988	-0.119	0.0152	*
2016	Chlor (µg/cm)	E9	44.9	44.7	0.197	0.800	NS	41.3	39.4	1.9	0.0575	+
2016	Chlor (µg/cm)	E10	42.9	44.4	-1.44	0.111	NS	38.0	42.2	-4.13	6.50E-05	***
2016	Chlor (µg/cm)	E11	43.2	43.8	-0.632	0.431	NS	39.3	41.1	-1.81	0.0419	*
2016	Shed (DAP)	E9	53.3	53.1	0.0785	0.708	NS	53.7	54.1	-0.345	0.186	NS
2016	Shed (DAP)	E10	53.0	53.6	-0.516	0.0154	*	53.6	54.0	-0.435	0.0919	+
2016	Shed (DAP)	E11	52.7	53.4	-0.657	0.00335	**	53.0	54.1	-1.15	2.00E-05	***
2016	Silk (DAP)	E9	53.9	54.1	-0.146	0.647	NS	54.2	55.3	-1.13	6.07E-04	***
2016	Silk (DAP)	E10	53.6	54.5	-0.869	0.00749	**	54.4	55.7	-1.29	7.46E-05	***
2016	Silk (DAP)	E11	53.8	54.0	-0.241	0.476	NS	53.9	55.1	-1.21	2.01E-04	***
2016	ASI (days)	E9	0.613	0.949	-0.336	0.272	NS	0.459	1.39	-0.935	0.00132	**
2016	ASI (days)	E10	0.496	0.797	-0.301	0.325	NS	0.640	1.73	-1.08	1.63E-04	***
2016	ASI (days)	E11	1.06	0.578	0.521	0.106	NS	1.06	0.946	0.115	0.679	NS
2016	Tot DW (g/pl)	E9	295	333	-38.3	0.00244	**	273	275	-1.89	0.815	NS
2016	Tot DW (g/pl)	E10	304	344	-39.3	0.00219	**	276	277	-1.27	0.873	NS
2016	Tot DW (g/pl)	E11	315	355	-39.5	0.00393	**	280	276	4.43	0.582	NS
2016	Seed DW (g/pl)	E9	139	153	-14.0	0.0627	+	125	118	7.43	0.325	NS
2016	Seed DW (g/pl)	E10	145	154	-8.48	0.266	NS	135	126	8.77	0.23	NS
2016	Seed DW (g/pl)	E11	136	161	-25.3	0.00221	**	126	130	-4.28	0.564	NS
2016	Seed no/pl	E9	381	413	-31.9	0.105	NS	362	329	32.4	0.198	NS
2016	Seed no/pl	E10	400	418	-18.2	0.367	NS	367	323	43.6	0.0784	+
2016	Seed no/pl	E11	396	439	-42.5	0.0485	*	355	352	3.02	0.904	NS
2016	100 seed wt (g)	E9	36.2	37.4	-1.20	0.163	NS	34.8	35.2	-0.399	0.661	NS
2016	100 seed wt (g)	E10	36.2	36.2	-0.0084	0.992	NS	36.7	35.4	1.30	0.147	NS
2016	100 seed wt (g)	E11	35.0	36.3	-1.29	0.152	NS	36.2	35.7	0.500	0.578	NS
2016	HI	E9	0.464	0.459	0.0051	0.732	NS	0.464	0.420	0.0438	0.0536	+
2016	HI	E10	0.475	0.444	0.0309	0.0494	*	0.482	0.423	0.0593	0.00864	**
2016	HI	E11	0.449	0.451	-0.0014	0.931	NS	0.458	0.457	4.66E-04	0.983	NS
